# Resistance to Diet-Induced Obesity and Associated Metabolic Perturbations in Haploinsufficient Monocarboxylate Transporter 1 Mice

**DOI:** 10.1371/journal.pone.0082505

**Published:** 2013-12-18

**Authors:** Sylvain Lengacher, Touria Nehiri-Sitayeb, Nadia Steiner, Lionel Carneiro, Céline Favrod, Frédéric Preitner, Bernard Thorens, Jean-Christophe Stehle, Laure Dix, François Pralong, Pierre J. Magistretti, Luc Pellerin

**Affiliations:** 1 Department of Physiology, University of Lausanne, Lausanne, Switzerland; 2 Laboratory of Neuroenergetic and Cellular Dynamics, Brain and Mind Institute, Ecole Polytechnique Fédérale de Lausanne, Lausanne, Switzerland; 3 Mouse Metabolic Evaluation Facility, Center for Integrative Genomics, University of Lausanne, Lausanne, Switzerland; 4 Mouse Pathology Facility, Institut Universitaire de Pathologie, Centre Hospitalier Universitaire Vaudois, Lausanne, Switzerland; 5 Service d’endocrinologie, diabétologie et métabolisme, Centre Hospitalier Universitaire Vaudois, Lausanne, Switzerland; University of Cordoba, Spain

## Abstract

The monocarboxylate transporter 1 (MCT1 or SLC16A1) is a carrier of short-chain fatty acids, ketone bodies, and lactate in several tissues. Genetically modified C57BL/6J mice were produced by targeted disruption of the *mct1* gene in order to understand the role of this transporter in energy homeostasis. Null mutation was embryonically lethal, but *MCT1* ^+/−^ mice developed normally. However, when fed high fat diet (HFD), *MCT1* ^+/−^ mice displayed resistance to development of diet-induced obesity (24.8% lower body weight after 16 weeks of HFD), as well as less insulin resistance and no hepatic steatosis as compared to littermate *MCT1* ^+/+^ mice used as controls. Body composition analysis revealed that reduced weight gain in *MCT1* ^+/−^ mice was due to decreased fat accumulation (50.0% less after 9 months of HFD) notably in liver and white adipose tissue. This phenotype was associated with reduced food intake under HFD (12.3% less over 10 weeks) and decreased intestinal energy absorption (9.6% higher stool energy content). Indirect calorimetry measurements showed ∼ 15% increase in O_2_ consumption and CO_2_ production during the resting phase, without any changes in physical activity. Determination of plasma concentrations for various metabolites and hormones did not reveal significant changes in lactate and ketone bodies levels between the two genotypes, but both insulin and leptin levels, which were elevated in *MCT1* ^+/+^ mice when fed HFD, were reduced in *MCT1* ^+/−^ mice under HFD. Interestingly, the enhancement in expression of several genes involved in lipid metabolism in the liver of *MCT1* ^+/+^ mice under high fat diet was prevented in the liver of *MCT1* ^+/−^ mice under the same diet, thus likely contributing to the observed phenotype. These findings uncover the critical role of MCT1 in the regulation of energy balance when animals are exposed to an obesogenic diet.

## Introduction

Short-chain monocarboxylates such as lactate or the ketone bodies ß-hydroxybutyrate and acetoacetate are metabolic substrates that play crucial roles in body energy homeostasis under various conditions (e.g. exercise or fasting). Indeed, lactate represents an important oxidative energy substrate for various tissues such as the heart [1], the brain [2] or the oxidative fibers of muscles [3]. Moreover, lactate is used by the liver for gluconeogenesis, as part of the Cori cycle [4]. Lactate also regulates both food intake and blood glucose levels by acting directly on fuel-sensitive neurons in the hypothalamus [5]–[7]. In parallel, ketone bodies produced during periods of fasting or under high fat diets [8], [9] are used as energy substrates by several tissues including the brain [10], the heart [11] and skeletal muscles [12]. Ketone bodies have also been shown to affect food intake [13] and body weight gain [14]. From a clinical point of view, it has been suggested that both lactate [15] and ketone bodies [16] metabolism could play an important role in the development of obesity although the underlying mechanisms remain obscure.

Interestingly, lactate and ketone bodies share common membrane transporters. A restricted group of carriers has been identified and its members are collectively known as monocarboxylate transporters or MCTs. They belong to the SLC16 protein family and consist of four H^+^-linked transporters termed MCT1-4 [17]. The first member, MCT1 (or SLC16A1), has the broadest tissue distribution. It is abundant in brain, heart, muscle, liver and kidney and it is also expressed in important tissues for energy homeostasis such as adipose tissue and gut [17]–[19]. At the cellular level, it can exhibit a specific and differential expression pattern. In skeletal muscle for example, it is particularly enriched in oxidative fibers [20]. MCT1 expression has been shown to be essential for monocarboxylate utilization in these different tissues and cell types. For the moment however, it is difficult to understand the importance of monocarboxylate exchange and utilization for the integrated regulation of body energy homeostasis without an appropriate *in vivo* model. In order to gain insight on this issue, a transgenic mouse model was generated in which the *mct1* gene was invalidated. The resulting metabolic phenotype of the haploinsufficient *MCT1^+/^* ^−^ mouse suggests a key role of this transporter in the regulation of body weight and fat accumulation, particularly upon exposure to an obesogenic diet.

## Materials and Methods

### Ethics statement

Animal experiments were performed in accordance with the Swiss animal welfare laws under the authorization n° VD 2252 from the Service de la consommation et des affaires vétérinaires du Canton de Vaud, Switzerland.

### Knockin targeting construct and generation of MCT1 knockout/LacZ knockin mice

**Figure 1:**
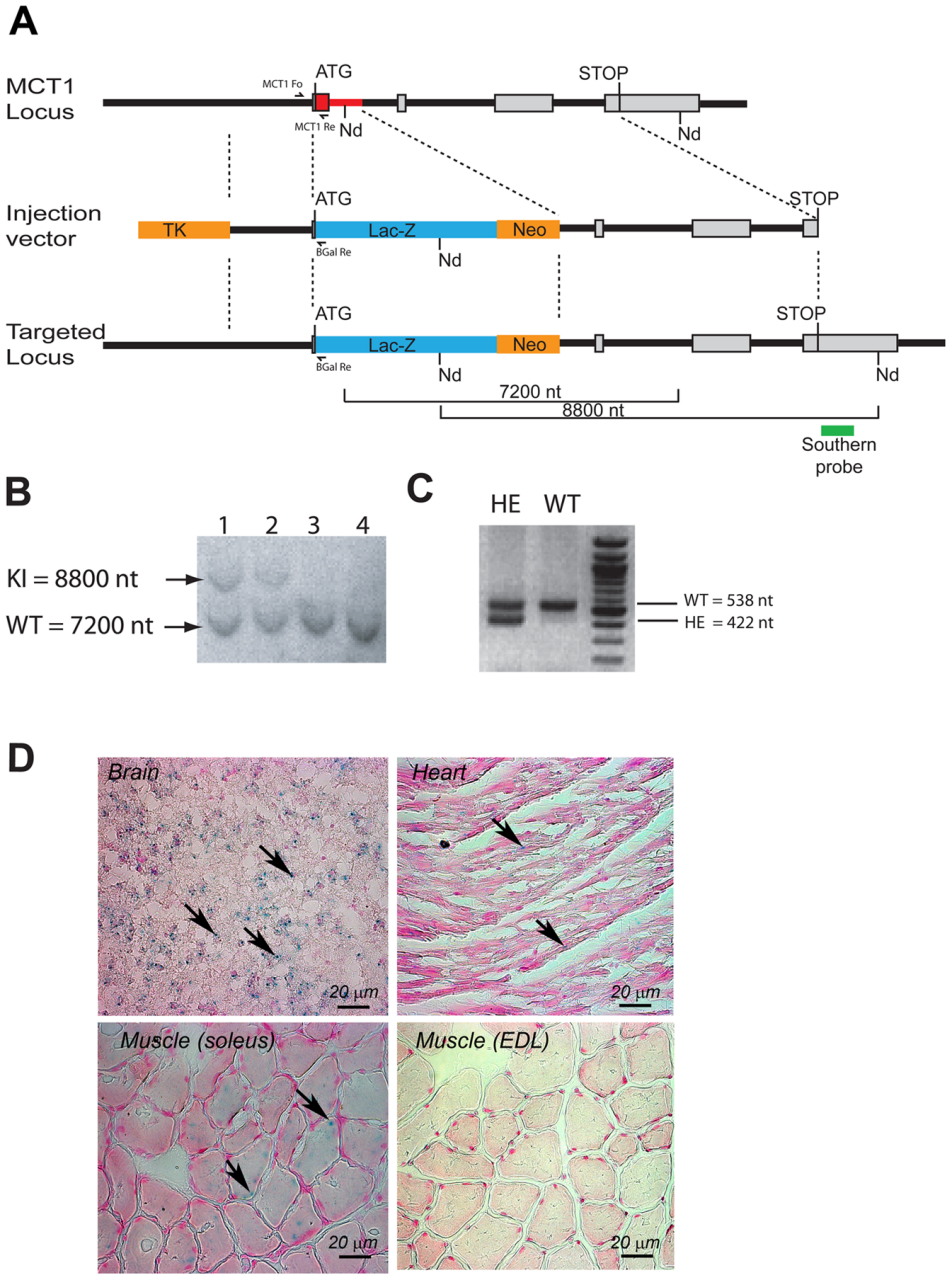
Gene targeting strategy to produce the *MCT1* knockout/ß-galactosidase knockin mouse. (**A**) Structure of the *MCT1* wildtype allele, the targeting vector, and the resulting *MCT1* knockout/ß-galactosidase knockin allele showing the targeted locus. Exons are represented as grey boxes except exon 1 that contains the translation initiation codon (ATG). The *mct1* gene sequence (640 bp) replaced by the fused LacZ/Neo gene sequence is indicated in red. TK, thymidine kinase gene for negative selection with Gancyclovir ; ß-Gal, the LacZ gene sequence coding for the enzyme ß-galactosidase ; Neo, neomycin resistance gene for positive selection with G418. (**B**) Southern blot performed with a 900 bp probe (green in A) on selected embryonic cell (ES) DNA digested with NdeI (Nd). ES 1 and 2 cell populations are showing a 7200 nt band corresponding to the wildtype (WT) allele and a 8800 nt band corresponding to the modified knockin (KI) allele due to a double recombination event. ES 3 and 4 cell populations did not undergo the double recombination event and only exhibit the 7200 nt band. (**C**) PCR genotyping strategy. PCR made with the primers MCT1 Fo, MCT1 Re and ßGal Re (see localization in A) present in the same reaction produces a fragment of 538 nt for wildtype (WT) animals, but also an additional fragment of 422 nt for heterozygote (HE) animals. (**D**) Histochemical detection of ß-galactosidase activity in selected tissues of *MCT1* ^+/LacZ^ mice. The blue color in cells reflects the presence of the ß-galactosidase enzyme and therefore the *mct1* promoter activity (arrows). Tissue counterstaining was done with nuclear fast red dye. EDL, *extensor digitorum longus*.

Heterozygote MCT1 knockout/LacZ knockin mice (referred to as *MCT1* ^+/−^ or *MCT1* ^+/LacZ^ mice) were generated by targeted homologous recombination. A targeting construct was made by fusing the thymidine kinase (TK) gene sequence with 1600 base pairs (bp) of the mouse genomic sequence (129/OlaHsd strain) ending with part of the first exon containing the translation initiation codon of the MCT1 gene (Fig. 1A). Then from the start codon, 640 bp of the MCT1 gene containing exon 1 and part of the first intron were replaced by the LacZ gene sequence fused with a neomycin (Neo) resistance gene sequence and put in frame with the MCT1 promoter. The targeting construct was completed with the following 5800 bp of the MCT1 genomic sequence starting from the middle of the first intron until the stop codon of MCT1 gene sequence. This construct was cloned in the pGEM-T-easy vector (Promega, Wallisellen, Switzerland). Before electroporation into mouse embryonic stem (ES) cells (from 129 OlaHsd strain), the vector was linearized by digestion with SacII (Roche Diagnostics, Rotkreuz, Switzerland). ES cells with proper sequence integration were selected by adding gancyclovir and G418 in ES cell culture medium for negative and positive selection, respectively [21]. A total of 704 neomycin resistant ES clones were tested by PCR for the presence of the targeting construct into the genome. Primer sequences were chosen to span the upstream genomic sequence serving for recombination. Four ES clones were found positive and tested further by Southern blot analysis. DNA was purified from the PCR positive clones, and digested with NdeI (Roche Diagnostics, Rotkreuz, Switzerland) before being processed by normal Southern blot procedure (Fig. 1B). A 900 bp genomic sequence, not contained in the targeting construct, was chosen as Southern probe to test ES DNA clones that have undergone a correct double recombination event. Cells from a positive ES clone were injected into 106 blastocysts (C57BL/6). Blastocysts were implanted into foster mothers. Among the 29 newborns obtained, 8 presented a chimeric fur (agouti pigmentation). Two chimeric males were crossed with C57BL/6 females. DNA extracted from F1 agouti (50% 129Ola, 50% C57BL/6) offsprings was tested by PCR and Southern blot for the presence of the modified allele. Electroporation in ES cells, selection of positive clones, transfer into blastocysts and implantation, as well as viable chimeric mouse selection were performed by genOway (Lyon, France, info@genoway.com). Animal breeding was performed by Charles River (L'Arbresle Cedex, France). F1 progeny carrying the transgene was backcrossed for 8 generations onto the C57BL/6 background. Subsequently, heterozygous MCT1 knockout/LacZ knockin males and females were crossed for characterization of all resulting genotypes. Mice were genotyped by PCR with a combination of 3 primers (ßGalRe GATTAAGTTGGGTAACGCCAG, MCT1Fo3962 GTGTCACCCAACACTGATAACAAGAG; MCT1Re4500 TGATACTTCACTGGTCGTTGCA), giving rise to a 535 bp wild type fragment and a 422 bp knockin fragment. The official name of the mutant mouse following the guidelines of the international committee of standardized genetic nomenclature for mice (http://www.informatics.jax.org/mgihome/nomen/gene.shtml) is: B6.129OlaHsd-MCT1tm(lacZ)Syle.

### Animals

*MCT1* ^+/−^ mice on the C57BL/6J genetic background were bred to produce heterozygous (*MCT1* ^+/−^) mice and wildtype (*MCT1* ^+/+^) littermate controls. Male mice were sent by Charles River (L’Arbresle Cedex, France) at 4 weeks of age. All animals were housed in cages placed in a controlled environment room, with a constant temperature of 20–22°C, relative moisture 50-60% and 12h light-dark cycle. Mice were allowed free access to food and water.

### ß-Galactosidase histological staining

Mouse tissue cryosections were put onto Superfrost Plus slides (Thermo scientific, Braunschweig, Germany) and allowed to dry at room temperature for 10 minutes. Then they were washed once with 1x Phosphate Buffer Saline (PBS) solution. An X-Gal staining solution (potassium ferricyanide (C_6_FeK_3_) 5 mM, potassium ferrocyanide (C_6_FeK_4_) 5 mM, 200 µl of X-Gal solution at 50 mg/ml (Promega, Wallisellen, Switzerland; cat N° V394A) in 10 ml PBS solution) was added onto tissue sections. The slides were incubated for 15 hours at 37°C. To avoid evaporation, slides were placed in a H_2_O saturated incubator. The box containing the slides was wrapped with aluminium foil to protect from light. Then tissue sections were washed once with 1x PBS solution, then fixed for 15 minutes with 4% paraformaldehyde solution and washed once with 1x PBS solution. Tissue sections were counterstained with nuclear fast red solution (Sigma, Buchs, Switzerland; cat N° N3020), then dehydrated and mounted with Eukitt solution (Kindler, Freiburg, Germany).

### Quantitative RT-PCR and Western blots

Mice were deeply anesthetized using sodium pentobarbital and transcardially perfused with cooled saline (0.9% NaCl + heparin 10 units/ml). Various tissues were rapidly collected and frozen in liquid nitrogen. Frozen tissues were ground into a fine powder using a mortar and pestle. To avoid RNA and protein degradation, we began our tissue collection by taking first pancreas and liver. Each tissue sample was divided in two to extract separately RNA and proteins. mRNA was extracted using the RNeasy mini kit (Qiagen, Basel, Switzerland; Cat N° 74104) following manufacturer’s instructions. Tissue samples were homogenized with the QIAshredder (Qiagen, Basel, Switzerland; Cat N° 79654) column before mRNA purification. For protein extraction, 40 mg of tissue powder were dissolved in buffer A (30 mM HEPES, 210 mM sucrose, 40 mM NaCl, 2 mM EGTA, 1% SDS, Protease inhibitors). Samples of protein extracts were then centrifuged at 12’000 RPM for 5 min. The protein supernatant was used for gel electrophoresis.

Quantification of mRNA was performed with the Nanodrop ND-1000 apparatus (Witec, Littau, Switzerland). One hundred ng of mRNA in a volume of 25 µl were reverse transcribed using the RT High Capacity RNA-to-cDNA Kit (Applied Biosystems, Rotkreuz, Switzerland). Quantitative real-time PCR analysis was performed with the Applied Biosystems 7900 (Applied Biosystems, Rotkreuz, Switzerland) Real-Time PCR System. The Taq polymerase master mix employed was the Power SYBR Green (Applied Biosystems, Rotkreuz, Switzerland). Primer sequences used for *MCT1* (NM_009196) mRNA quantification were mMCT1Fo1361 AATGCTGCCCTGTCCTCCTA mMCT1Re1441 CCCAGTACGTGTATTTGTAGTCTCCAT, and for *ß-actin* (NM_007393) mActinßFo31 GCTTCTTTGCAGCTCCTTCGT mActinßRe94 ATATCGTCATCCATGGCGAAC. ß-actin gene was used as endogenous control. To evaluate if the *ß-actin* gene was suitable as endogenous control, we performed quantification using *ß-actin*, *cyclophilin A* (NM_008907) mCycloFo343 CAAATGCTGGACCAAACACAA mCycloRe413 GCCATCCAGCCATTCAGTCT, and *HPRT* (NM_013556) mHPRTFo514 GCAGTACAGCCCCAAAATGG, mHPRTRe598 AACAAAGTCTGGCCTGTATCCAA as other endogenous controls and used the GeNORM method [22] to quantify MCT1 expression in different tissues (data not shown). *ß-actin* was chosen for the studies as we obtained similar results when we analyzed our data either with GeNORM procedure or using *β-actin* gene only as endogenous control. All primer pairs were designed to overlap exon-exon junction avoiding contamination signal from eventual genomic DNA. Primers specificity and efficiency was tested with various amounts of cDNA. All samples were analyzed in triplicates. Cycle threshold (C_T_) values obtained with SDS2.3 software (Applied Biosystems, Rotkreuz, Switzerland) were imported in an Excel sheet for calculation.

For the study of hepatic gene expression, Messenger RNA from liver were extracted using RNeasy mini kit (Qiagen) following manufacturer’s instruction. Quantification of mRNA was performed using the Nanodrop 1000. One hundred of mRNA were transcribed using TaqMan reverse transcription reagents (Cat. N° N808-0234, Applied Biosystems). Quantitative real-time PCR was performed on the Viia7 Real Time PCR system (Applied Biosystems). The Taq polymerase master mix employed was the Power SYBR Green (Applied Biosystems). 18s was chosen as endogenous control and data analysis was performed using the 2^−ΔΔCT^ method. The primers used for each target gene are indicated in Table 1.

**Table 1:** Sets of primers used for qRT-PCR of genes involved in lipid metabolism.

Genes			Sequence	
**ACC1**	forward	5′-	TGGTGCAGAGGTACCGAAGTG	-3′
	reverse	5′-	CTGCGGCAGCAGATCCAT	-3′
**ACC2**	forward	5′-	AACTCCCTGCCAAGCTCATG	-3′
	reverse	5′-	GCGGCTGTCCAGTTGGTTT	-3′
**FAS**	forward	5′-	AGGCTGGGCTCTATGGATTA	-3′
	reverse	5′-	AAAAGGAGGCGTCGAACTTG	-3′
**DGAT1**	forward	5′-	TTCCGCCTCTGGGCATT	-3′
	reverse	5′-	AGAATCGGCCCACAATCCA	-3′
**DGAT2**	forward	5′-	AGTGGCAATGCTATCATCATCGT	-3′
	reverse	5′-	AAGGAATAAGTGGGAACCAGATCA	-3′
**ADRP**	forward	5′-	GACCTTGTGTCCTCCGCTTAT	-3′
	reverse	5′-	CAACCGCAATTTGTGGCTC	-3′
**HSL**	forward	5′-	ACGGCGGCTGTCTAATGTC	-3′
	reverse	5′-	AAACTACGGTATCCGTTGGCT	-3′
**FAT/CD36**	forward	5′-	TCCCCCTACTAGAAGAAGTGGG	-3′
	reverse	5′-	TCCAACAGATTGGTTTCGTTC	-3′
**CPT1**	forward	5′-	CTCCGCCTGAGCCATGAAG	-3′
	reverse	5′-	CACCAGTGATGATGCCATTCT	-3′
**LCAD**	forward	5′-	TGGCATCAACATCGCAGAGA	-3′
	reverse	5′-	ACGCTTGCTCTTCCCAAGTAAC	-3′
**ACO**	forward	5′-	CCAATGCTGGTATCGAAGAATG	-3′
	reverse	5′-	GGAATCCCACTGCTGTGAGAA	-3′
**UCP2**	forward	5′-	TCCCCTGTTGATGTGGTCAA	-3′
	reverse	5′-	CAGTGACCTGCGCTGTGGTA	-3′
**SREBP1c**	forward	5′-	GGAGCCATGGATTGCACATT	-3′
	reverse	5′-	GGCCCGGGAAGTCACTGT	-3′
**PPARgamma**	forward	5′-	GGAAGACCACTCGCATTCCTT	-3′
	reverse	5′-	GTAATCAGCAACCATTGGGTCA	-3′
**18s**	forward	5′-	GTAACCCGTTGAACCCCATT	-3′
	reverse	5′-	CCATCCAATCGGTAGTAGCG	-3′

For western blots, tissue samples were mixed (1:1) with 2 x SDS gel loading buffer (100 mM Tris-Cl pH 6.8, 200 mM dithiothreitol, 4% SDS, 0.1% bromophenol blue, 10% glycerol). Before loading on gel, samples were heated at 95°C for 5 minutes. Ten micrograms of protein were loaded onto Invitrogen NuPAGE 10% gel (Invitrogen, Basel, Switzerland; Cat N° NP0301BOX). Gels were run at 100 Volts and at 4°C. Proteins were transferred from gels on

Protran BA83 Nitrocellulose membranes (Whatman, Bottmingen, Switzerland; ref. 84261514) using the following transfer buffer: 0.4 M glycine, 0.25 M Tris-base pH 6.8, 20% methanol. Transfers were completed by application of 75 Volts during 80 min at 4°C. Membranes were then left for 1 hour at ambient temperature in 1x PBS containing 0.1% Tween 20 and 2% of blocking reagent from ECL advance WB detection kit (GE Healthcare, Piscataway, NJ, USA). The primary antibody was left overnight at 4°C in the same blocking buffer diluted at 1∶2000 for anti-MCT1 (Novus Biologicals, Littleton, CO, USA; cat. n° H000006566-B01P) and 1∶20000 for anti-ß-actin (Sigma, Buchs, Switzerland; cat. n° A5441). After washing, the secondary antibody anti-mouse (1∶10000) horseradish peroxidase conjugate (GE Healthcare, Piscataway, NJ, USA) was applied for one hour at ambient temperature. Luminescence detection was performed by following manufacturer’s instructions (GE Healthcare, Piscataway, NJ, USA) for the ECL advance WB detection kit. Images were acquired using the Chemidoc XRS system (Bio-Rad, Reinach, Switzerland). Band intensities quantification was done with the Quantity One software (Bio-Rad, Reinach, Switzerland).

### Histological analysis

Animals were anesthetized with isoflurane before being killed by cervical dislocation. An incision in the skin was made from rectum to the oesophagus and the entire animal was put in a buffered formol fixating solution for 24 hours. Various tissues and organs were dissected, paraffin-embedded with a Leica ASP300S tissue processor (Leica, Heerbrug, Switzerland) and 3 µm tissue sections prepared with a Microm HM 335 E microtome (Thermo Scientific, Walldorf, Germany). Each section was stained with hematoxylin and eosin standard procedure, mounted on glass slides and examined with a Nikon Eclipse 80i microscope (Nikon AG, Egg, Switzerland) using brightfield optics at 40x magnification. Digital images of representative sections were made for illustrative purposes. For subcutaneous, perigonadal and inguinal white adipose tissues, each one was dissected immediately after sacrifice of the animal and fixed in a 4% paraformaldehyde solution for 24 hours. After being paraffin-embedded, 40 µm sections were prepared with a microtome and stained with hematoxylin and eosin. Sections mounted on glass slides were examined in the same manner as the other tissue sections.

### Morphological, behavioural and functional phenotyping

A modified primary *SHIRPA* protocol was performed on *MCT1* ^+/+^ and *MCT1* ^+/−^ mice [23]. Briefly, mice were observed during a five minutes period in a jar for spontaneous activity, body position, respiration and tremor. Then mice were transferred to an arena (a clean rat cage) where transfer arousal, palpebral closure, piloerection, gait, pelvic elevation, tail elevation, touch escape, postural passivity, and shock hearing were monitored. The following parameters were evaluated above the arena by taking the animal by the tail: trunk curl, limb grasping, visual placing, grip strength. The animal was then held by the tail but kept on the ground to check pinna reflex, corneal reflex, toe pinch and skin colour. The animal was held firmly to measure the body length, lacrimation, salivation, provoked biting, abdominal tone, coat colour, and pressure resistance on the left leg. Other tests that were performed include: wire manoeuvre, reflex positioning, negative geotaxis. During testing, fear, irritability, aggression, scream and coat tenseness were rated. The coat hanger test, measured grip strength (Harvard Apparatus, Les Ulis, France) and a rotarod test with a fix speed (Harvard Apparatus, Les Ulis, France) were performed. Body temperature was also recorded.

### Diet studies/body weight evaluation

*MCT1* ^+/−^ and *MCT1* ^+/+^ littermates were individually housed and received either a normal chow diet (NC) containing 4.5% fat (Provimi Kliba, Penthalaz, Switzerland; Cat n° 3436) or a high fat diet (HFD) in which 54.8% of calories are provided by fat (Harlan Teckland, Oxon, UK; Cat N° TD.93075) with unlimited access to food and water from five weeks of age. Body weight was determined weekly.

### Intraperitoneal glucose and insulin tolerance tests

After a 15 hours overnight fasting or a 5 hours fasting respectively, mice received D-glucose (1 mg/g of body weight; Applichem Gmbh, Darmstadt, Germany) or insulin (0.3 mU/g of body weight ; Novo Nordisk A/S, Bagsvaerd, Denmark) by intraperitoneal injection. Glucose concentrations were measured at 0, 15, 30, 60 and 120 min after glucose or insulin injection in blood samples obtained from tail-tip bleedings, using a Glucometer (Glucotrend Premium, Boehringer, Ingelheim, Germany). Areas under the curve (AUCs) were measured from 0 to 120 minutes, after subtraction of basal glycemia from each time point.

### Body composition

Body composition (fat mass) was determined on isoflurane anaesthetized *MCT1* ^+/−^ and *MCT1* ^+/+^ mice using an Echo MRI analyzer (Whole Body Composition Analyzer, Echo medical systems, Houston, TX, USA). Each mouse was weighed before body composition analysis. Each measurement was performed twice, and the mean value was reported. Moreover, subcutaneous, perigonadal, and inguinal white adipose tissues as well as the liver were removed at the end of the experiment and weighed prior to be fixed for histological analysis.

### Food intake

Mice were provided weekly with food that was weighed and recorded. Food consumption was manually monitored weekly for each mouse by subtracting the amount of food remaining (food compartment + spillage in cage bedding). Individual body weight was also determined weekly.

### Stool energy content

Feces were collected, dried to a constant weight (± 0.001 g) at 60°C, and mean energy content (kJ/g) was determined for each mouse using a bomb calorimeter (IKA C200, Staufen, Germany).

### Physical activity and indirect calorimetry

Spontaneous locomotor activity (horizontal and vertical movements), oxygen consumption, and CO_2_ production were simultaneously determined for 16 mice per experiment, by indirect, open-circuit calorimetry, in an Oxymax Metabolic Chamber system (Columbus Instruments, Columbus, OH, USA). A total of 102 measurements/mouse/24 hours was recorded. Adult mice (8–14 weeks old for NC and 14–19 weeks old for HFD) were monitored for 120 hours but only the last 72 hours of each experimental run was used for data analysis (n = 8 for both genotypes).

### Blood analysis

*MCT1* ^+/+^ (n = 5) and *MCT1* ^+/−^ (n = 7) mice maintained on normal chow diet and *MCT1* ^+/+^ (n = 4) and *MCT1* ^+/−^ (n = 8) mice maintained on high fat diet for 3 months were anesthetized with pentobarbital by i.p. injection and blood samples were collected from cardiac puncture. Plasma chemistry was analyzed using the Roche/Hitachi 902 robot system (Roche). Plasma insulin and leptin were determined using the mouse metabolic Milliplex kit (MMHMAG-44k, Merck Millipore) following manufacturer’s instructions and were quantified on a Luminex MAGPIX (Luminex Corporation). All samples were measured in duplicate. Results were analyzed with the Milliplex analyst software (ver. 3.5.5.0, Merck Millipore).

### Statistics

All values are expressed as mean ± SEM. Statistical analyses were performed using either (where appropriate) a Student’s *t*-test or an analysis of variance (ANOVA) followed by a Tukey HSD or Bonferroni *post hoc* test with the following levels of significance: * = *P*<0.05, ** = *P*<0.01 and *** = *P*<0.001.

## Results

### *MCT1* ^−/−^ mice die at an early embryonic stage but *MCT1* ^+/−^

### mice are viable

A transgenic mouse was produced by disruption of the *mct1* locus. Replacement of a 640 bp sequence including the first exon within the *mct1* gene by a sequence coding for ß-galactosidase (LacZ gene) in frame with the *mct1* promoter was obtained by homologous recombination (Fig. 1A). Successful recombination was verified by Southern Blot on selected embryonic stem (ES) cell DNA. The presence of a 8800 nt band after digestion of DNA with the restriction enzyme NdeI (Nd) indicated the presence of the modified allele in some ES cell populations (Fig. 1B). Identification of transgenic animals was performed by PCR using specific primers that gave rise to a 422 nt band for the modified allele and a 538 nt band for the wildtype allele (Fig. 1C). No homozygous animal (referred to as *MCT1* **^−^** ^/**−**^ or *MCT1* ^LacZ/LacZ^) was ever identified among all newborns that were analyzed. Attempts to determine whether homozygous embryos died before birth suggested that their development must have been halted quite early as no homozygote embryo was ever observed. Among newborns, the ratio of males vs. females was the same. Only male mice were included in the experiments reported herein.

In order to verify that the transgene introduced in the *mct1* locus was properly inserted and expressed, sections of tissues known for either high (e.g. brain) or low (e.g. EDL muscle) MCT1 expression were incubated with the ß-galactosidase substrate X-gal. Blue deposits indicating the presence of the active enzyme could be observed in the brain, the heart and the soleus muscle of *MCT1* ^+/LacZ^ mice while it was not detected in the *extensor digitorum longus* (EDL) muscle (Fig. 1D). No staining was seen in tissues from *MCT1* ^+/+^ animals (data not shown).

**Figure 2:**
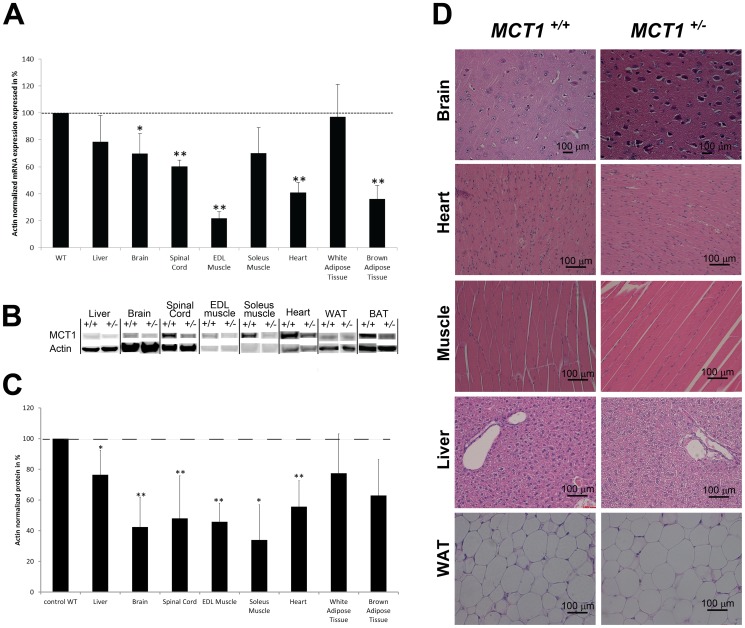
MCT1 mRNA and protein expression levels in tissues from *MCT1* ^+/+^ vs. *MCT1* ^+/−^ mice and comparative histological analysis. (**A**) Quantitative RT-PCR analysis of relative MCT1 mRNA levels in tissues collected from *MCT1^+/^* ^−^ and *MCT1^+/+^* mice. Values are expressed as percentage and represent the mean normalized MCT1 mRNA level in *MCT1^+/^* ^−^ mouse tissue compared to the mean normalized MCT1 mRNA level in *MCT1^+/+^* mouse tissue ± SEM, n = 6. ß-actin mRNA was used as reference. Statistical analysis was performed using the Student’s t-test. Asterisks indicate a significant difference between heterozygote and wildtype tissues. *P<0.05, ** *P*<0.01. (**B**) Representative western blot performed on protein extracts from selected tissues of *MCT1^+/+^* and *MCT1^+/^* ^−^ mice. ß-actin was used as reference for quantification. (**C**) Quantitative analysis of MCT1 protein expression. Values are expressed as percentage, and represent the mean normalized MCT1 protein level of *MCT1^+/^* ^−^ mice compared to the mean normalized MCT1 protein level for *MCT1^+/+^* mice ± SEM, n = 6. Statistical analysis was performed using the Student’s *t*-test. Asterisks indicate a significant difference between heterozygote and wildtype tissues. *P<0.05, ** *P*<0.01. (**D**) Comparative histological analysis of selected tissues from *MCT1^+/+^* and *MCT1^+/^* ^−^ mice. Sections from brain (cortex), heart, skeletal muscle (mixed type), liver, and white adipose tissue (WAT) of both *MCT1^+/+^* and *MCT1^+/^* ^−^ mice were stained with hematoxylin/eosin and examined under light microscopy at 40x magnification. Calibration bar, 100 µm.

Assessment of MCT1 mRNA expression in selected tissues was performed in both *MCT1* ^+/**−**^ and *MCT1* ^+/+^ mice and reported as relative expression. A significant reduction of MCT1 mRNA levels was observed in brain, spinal cord, EDL and soleus muscles, heart as well as brown adipose tissue (BAT) of *MCT1* ^+/**−**^ mice compared to *MCT1* ^+/+^ animals (Fig. 2A). Decrease in expression ranges from 20% (liver) to almost 80% (EDL muscle). Determination of MCT1 protein levels in the same tissues by western blot (Fig. 2B) revealed a significant reduction of approximately 50% in brain, spinal cord, EDL and soleus muscles, heart as well as BAT of *MCT1* ^+/**−**^ mice compared to *MCT1* ^+/+^ mice (Fig. 2C). Histological analysis of several tissues (Fig. 2D and Fig. S1) did not reveal striking structural differences between *MCT1* ^+/+^ and *MCT1* ^+/**−**^ mice. A further morphological, behavioral and functional assessment of the *MCT1* ^+/**−**^ mouse phenotype was performed using the SHIRPA protocol [23]. Except for the body length (nose-to-anus), other parameters were within normal range (Table S1).

### *MCT1* ^+/−^ mice exhibit resistance to diet-induced obesity as well as to associated glucose intolerance and insulin resistance

**Figure 3:**
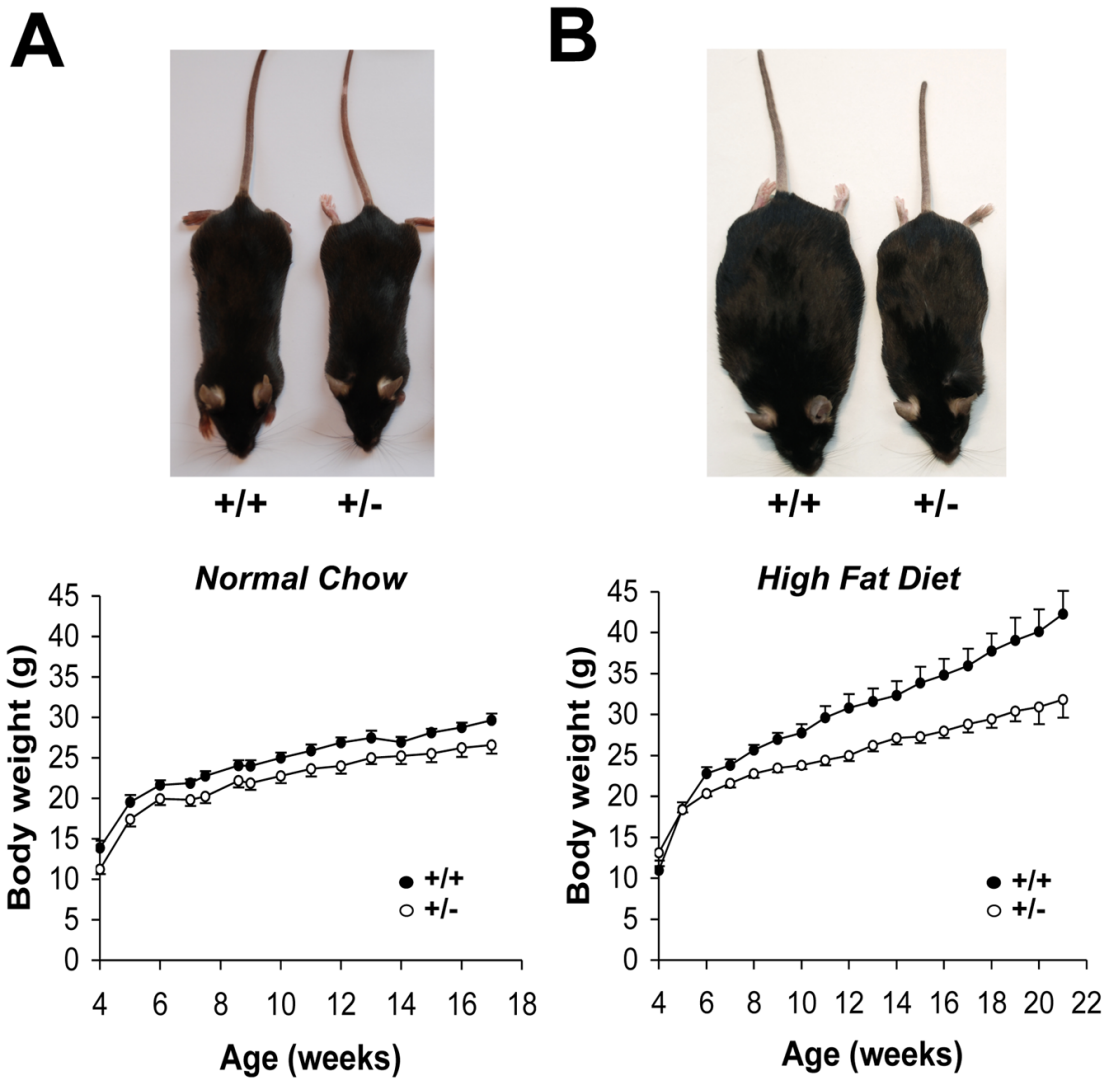
Body weight gain for *MCT1* ^+/+^ and *MCT1* ^+/−^ mice fed a normal chow or high fat diet. (**A**) Photograph of representative *MCT1* ^+/+^ and *MCT1* ^+/−^ mice fed with a normal chow diet for 12 months (upper panel). Weight gain over time for *MCT1* ^+/+^ and *MCT1* ^+/−^ mice fed with a normal chow diet (lower panel). (**B**) Photograph of representative *MCT1* ^+/+^ and *MCT1* ^+/−^ mice fed with a high fat diet for 12 months (upper panel). Weight gain over time for *MCT1* ^+/+^ and *MCT1* ^+/−^ mice fed starting at 5 weeks of age with a high fat diet (lower panel). +/+, *MCT1* ^+/+^; +/-, *MCT1* ^+/−^. Filled circles, *MCT1* ^+/+^; open circles, *MCT1* ^+/−^. Results are mean ± SEM, n = 7.

*MCT1* ^+/**−**^ mice were almost undistinguishable morphologically from *MCT1* ^+/+^ mice (Fig. 3A, upper panel). Comparison of body weight evolution under normal chow diet (NC) revealed a similar rate of body weight gain between *MCT1* ^+/+^ and *MCT1* ^+/**−**^ mice (Fig. 3A, lower panel). Although body weight of *MCT1* ^+/**−**^ mice was slightly lower (due to a slightly smaller body length), this initial difference was maintained over the entire period studied. When fed a high fat diet (HFD), *MCT1* ^+/+^ mice gained considerable weight and became obese (Fig. 3B, upper panel). In contrast, *MCT1* ^+/**−**^ mice fed the same diet remained leaner. An evaluation of body weight gain over time for mice put under HFD starting at 5 weeks of age clearly shows that *MCT1* ^+/**−**^ mice increased their body weight at a much lower rate than *MCT1* ^+/+^ mice (Fig. 3B, lower panel). After 16 weeks under HFD, the difference in body weight reached on average 24.8% less for *MCT1* ^+/**−**^ mice compared to *MCT1* ^+/+^ mice (31.8±2.2 g vs. 42.3±2.8 g respectively, *P*<0.05).

**Figure 4:**
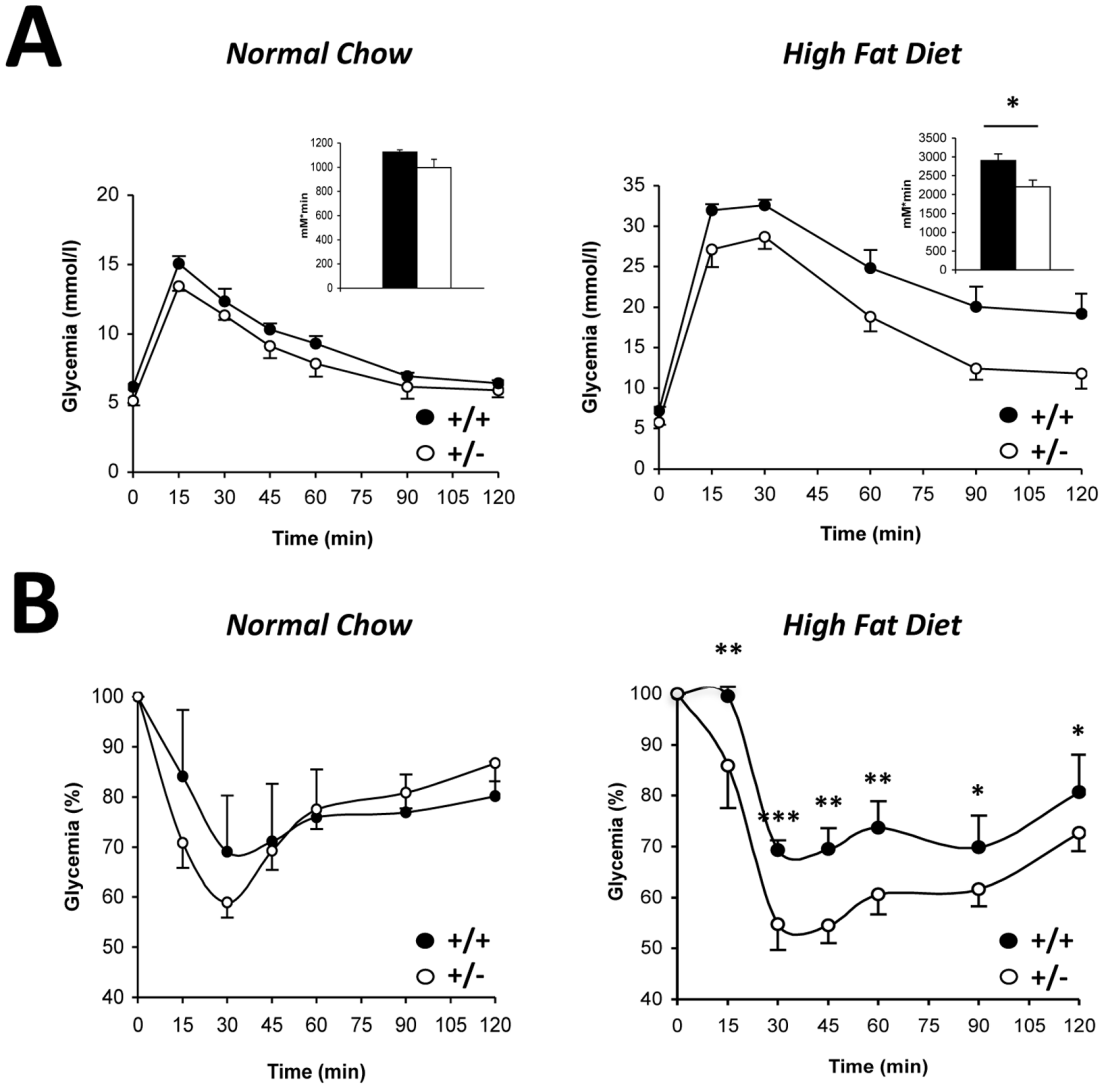
Glucose homeostasis in *MCT1* ^+/+^ and *MCT1* ^+/−^ mice fed a normal chow or high fat diet. (**A**) Intraperitoneal glucose tolerance test in *MCT1* ^+/+^ and *MCT1* ^+/−^ mice fed with a normal chow (left panel) or with a high fat diet (right panel) for 8 weeks. Inset: Quantitative analysis of integrated glycemic responses (as area under the curve or AUC). Filled bar, *MCT1* ^+/+^ mice. Open bar, *MCT1* ^+/−^ male mice (**B**) Intraperitoneal insulin tolerance test in *MCT1* ^+/+^ and *MCT1* ^+/−^ mice fed with a normal chow (left panel) or with a high fat diet (right panel) for 8 weeks. Filled circles, *MCT1* ^+/+^; open circles, *MCT1* ^+/−^. Results are mean ± SEM, n = 5–9. Asterisks represent a statistically significant difference between *MCT1* ^+/+^ and *MCT1* ^+/−^ mice evaluated using a Student’s *t*-test. *P<0.05, **P<0.01, ***P<0.001.

An important consequence of obesity is the development of insulin resistance. To evaluate this parameter, *MCT1* ^+/**−**^ and *MCT1* ^+/+^ mice fed NC or HFD were metabolically challenged. An intraperitoneal glucose tolerance test (IpGTT) was performed on 13 weeks-old *MCT1* ^+/**−**^ and *MCT1* ^+/+^ mice previously fed for 8 weeks either NC or HFD (Fig. 4A). Blood glucose levels under fasting conditions prior to glucose load (0 minute) were similar between *MCT1* ^+/**−**^ and *MCT1* ^+/+^ mice (5.6±0.3 vs. 5.9±0.2 mM respectively, *P* = 0.393 for NC condition; 5.3±0.2 vs. 6.3±0.2 mM respectively, *P* = 0.084, for HFD condition). The glycemic excursion in response to a glucose load was not different between *MCT1* ^+/**−**^ and *MCT1* ^+/+^ mice when fed NC (Fig. 4A, left panel; Inset: area under the curve  =  996±69 vs. 1127±16 mM/minute respectively, *P* = 0.145). In contrast, when fed HFD, the glycemic excursion was lower in *MCT1* ^+/**−**^ mice compared to *MCT1* ^+/+^ mice, indicating higher glucose tolerance (Fig. 4A, right panel; Inset: area under the curve  =  2208±178 vs. 2900±179 mM/minute respectively, *P*<0.05).

Insulin sensitivity was investigated 1 week later with an insulin tolerance test (ITT) on the same *MCT1* ^+/**−**^ and *MCT1* ^+/+^ mice previously fed either NC or HFD (Fig. 4B). The glycemic drop following insulin administration did not differ between *MCT1* ^+/**−**^ and *MCT1* ^+/+^ mice under NC (Fig. 4B, left panel). However, when the experiment was performed on mice fed HFD, the insulin-induced glycemic drop was more pronounced for *MCT1* ^+/**−**^ mice than for *MCT1* ^+/+^ mice indicating a better insulin sensitivity (Fig. 4B, right panel). Moreover, the difference in blood glucose levels remained between the two groups of mice for the duration of the experiment (120 minutes).

### Less fat accumulation in liver and white adipose tissue accounts for lower body weight gain in *MCT1* ^+/−^ mice under HFD

**Figure 5:**
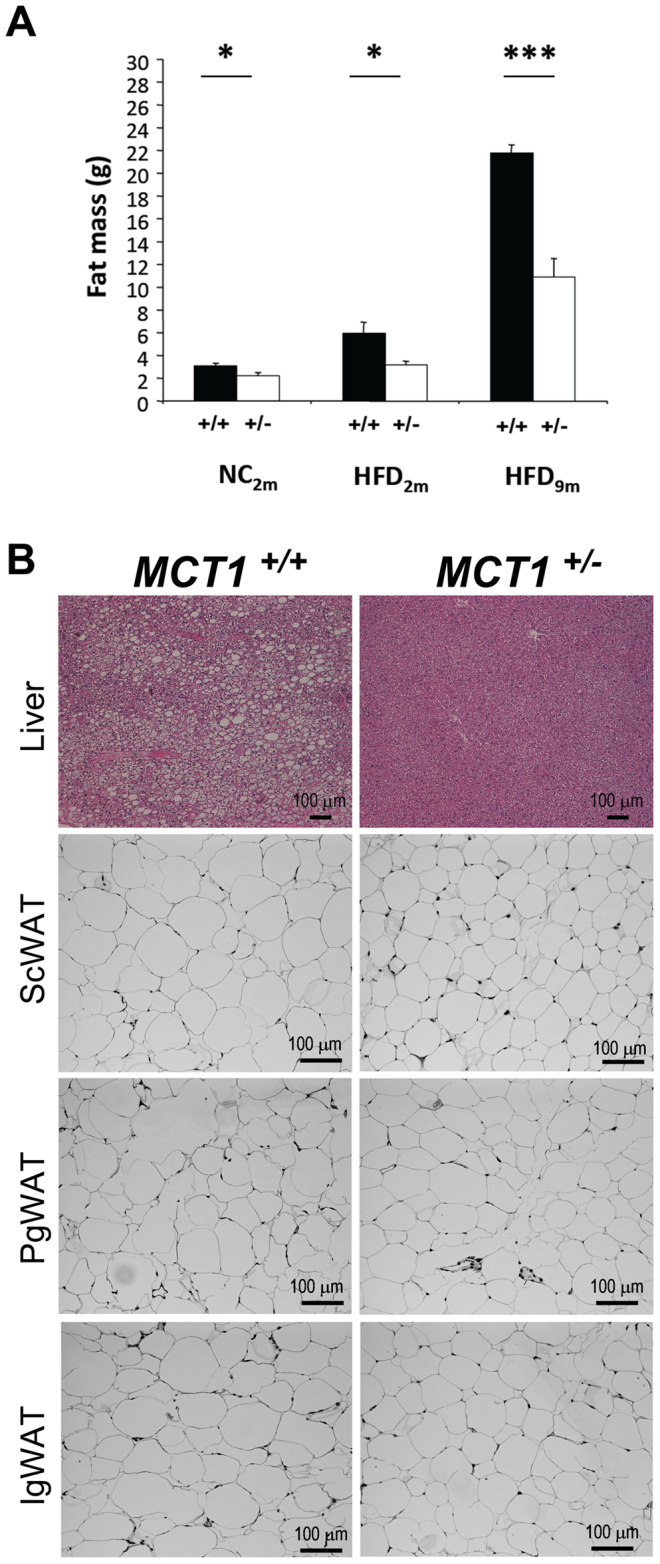
Fat mass accumulation and distribution in *MCT1* ^+/+^ and *MCT1* ^+/−^ mice fed a normal chow or high fat diet. (**A**) Fat mass changes in *MCT1* ^+/+^ and *MCT1* ^+/−^ mice fed a normal chow or a high fat diet for different periods of time. Results are mean ± SEM, n = 4-9. Asterisks represent a statistically significant difference between *MCT1* ^+/+^ and *MCT1* ^+/−^ mice evaluated using a Student’s *t*-test. *P<0.05, ***P<0.001. +/+, *MCT1* ^+/+^; +/-, *MCT1* ^+/−^; NC, normal chow; HFD, high fat diet; 2 m, 2 months; 9 m, 9 months. (**B**) Comparative histological analysis of liver and distinct adipose tissues of *MCT1* ^+/+^ and *MCT1* ^+/−^ mice fed a high fat diet for 11 months. ScWAT, subcutaneous white adipose tissue; PgWAT, perigonadal white adipose tissue; IgWAT, inguinal white adipose tissue. Calibration bar, 100 µm.

An analysis of body composition (to determine fat mass) by magnetic resonance proton spectroscopy (EchoMRI) was performed on *MCT1* ^+/**−**^ and *MCT1* ^+/+^ mice previously fed either NC or HFD (Fig. 5A). Results revealed that the body fat content of *MCT1* ^+/**−**^ mice is slightly but significantly lower than in *MCT1* ^+/+^ mice when fed NC for 2 months (2.23±0.27 g vs. 3.08±0.24 g respectively; *P*<0.05). When fed with HFD for 2 months, fat mass increased in both genotypes but less in *MCT1* ^+/**−**^ than in *MCT1* ^+/+^ mice (3.19±0.32 g vs. 5.94±0.98 g respectively; *P*<0.05). Expressed as percentage of total body weight, *MCT1* ^+/**−**^ mice exhibited 12.84±1.06% body fat while *MCT1* ^+/+^ mice had 19.41±2.31%. Mice fed during 9 months under HFD showed an important difference in fat mass between genotypes that reached more than 10 g (10.9±1.6 vs. 21.8±0.7 g for *MCT1* ^+/**−**^ and *MCT1* ^+/+^ respectively; *P*<0.001). Reported to body weight, fat represented 29.2±3.0% in *MCT1* ^+/**−**^ mice and 42.0±0.5% for *MCT1* ^+/+^ mice.

To gain further insight about alterations of body composition, *MCT1* ^+/+^ and *MCT1* ^+/**−**^ mice fed HFD for 11 months were dissected to collect several organs and tissues. Visual inspection revealed a difference in size of the liver and adipose tissue. Weighing confirmed that liver from *MCT1* ^+/+^ mice was almost 40% heavier (Table 2). When comparing adipose tissue from different locations, some differences also emerged (Table 2). Subcutaneous and perigonadal white adipose tissues (WAT) from *MCT1* ^+/**−**^ mice weigh significantly less. In contrast, no difference was observed for the inguinal WAT. Consistent with the visual inspection, observation of stained liver sections from *MCT1* ^+/+^ mice revealed an important hepatic steatosis (Fig. 5B, upper left panel). In contrast, liver from *MCT1* ^+/**−**^ mice appeared completely normal (Fig. 5B, upper right panel). A comparison of sections from subcutaneous WAT between *MCT1* ^+/**−**^ and *MCT1* ^+/+^ mice suggests that adipocytes are smaller in *MCT1* ^+/**−**^ mice (Fig. 5B). No such difference was detected for perigonadal and inguinal WAT (Fig. 5B). Other tissues from *MCT1* ^+/**−**^ mice maintained on HFD appeared normal (Fig. S2).

**Table 2:** Body, liver and white adipose tissues mass of HFD fed mice.

	MCT1^+/+^ (7)	MCT1^+/−^ (4)
**Body weight (g)**	55.2±1.0	44.3±2.6***
**Liver (g)**	4.27±0.20	2.65±0.39**
**ScWAT (g)**	1.87±0.14	1.16±0.13**
**PgWAT (g)**	3.84±0.24	2.98±0.25*
**IgWAT (g)**	0.69±0.06	0.64±0.11

Results are expressed as mean ± SEM ; values within parentheses indicate number of mice. *P<0.05, **P<0.01, ***P<0.001 by Student’s *t*-test. ScWAT, Subcutaneous white adipose tissue; PgWAT, Perigonadal white adipose tissue; IgWAT, Inguinal white adipose tissue.

### Lower food intake and intestinal absorption combined with higher metabolic rate contribute to lesser body weight gain in *MCT1* ^+/−^ mice under HFD

**Figure 6:**
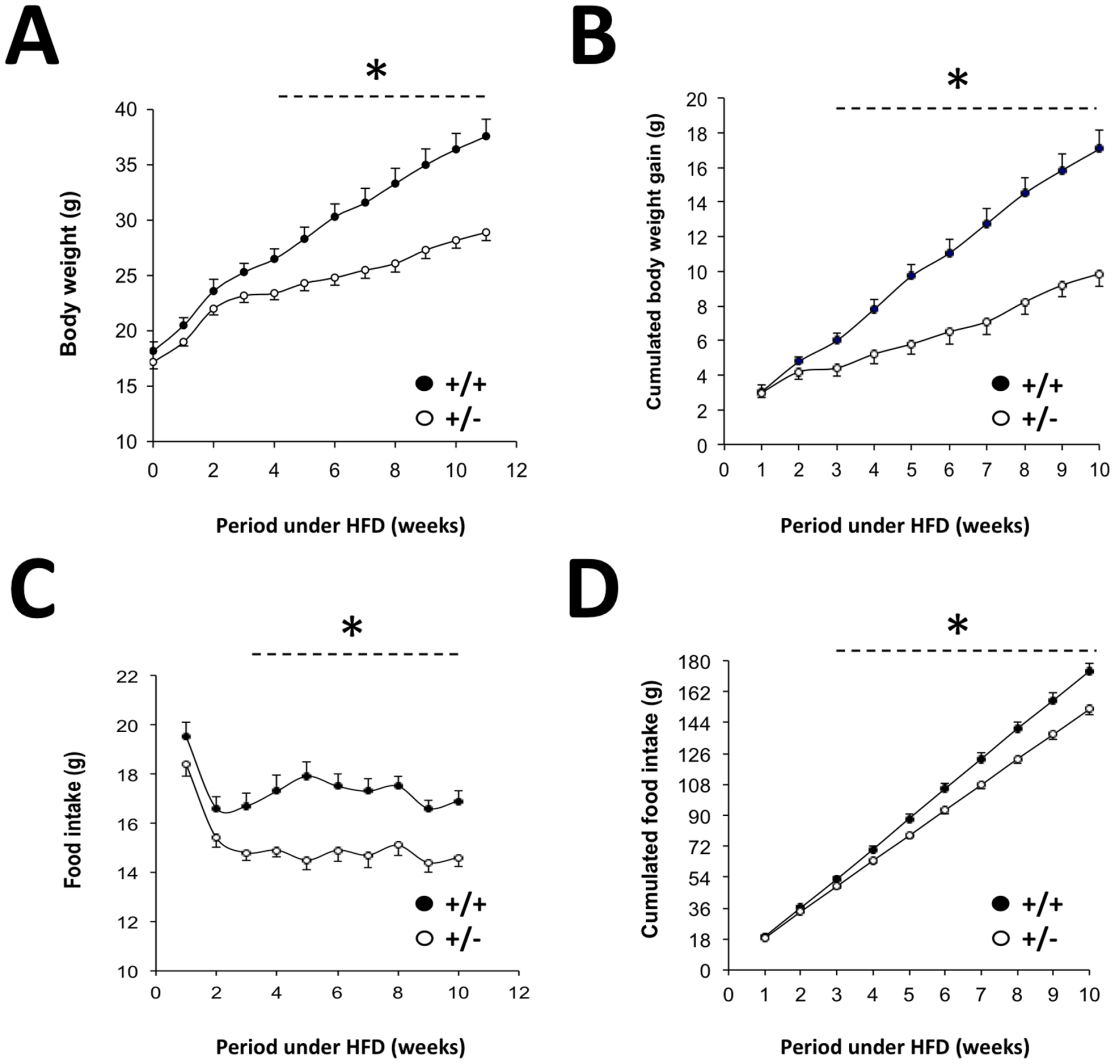
Body weight gain and food intake for *MCT1* ^+/+^ and *MCT1* ^+/−^ mice fed high fat diet. (**A**) Evolution with time of body weight (**B**) Cumulative body weight gain (**C**) Weekly measured food intake (**D**) Cumulative food intake. Results represent mean ± SEM, n = 10–11. Filled circles, *MCT1* ^+/+^ mice; open circles, *MCT1* ^+/−^ mice. The asterisk represents a statistically significant difference between *MCT1* ^+/+^ and *MCT1* ^+/−^ mice evaluated using a Student’s *t*-test for each pair of data under the dotted line. *P<0.05.

Modifications of energy intake and/or expenditure could explain the differences in weight gain observed between *MCT1* ^+/**−**^ and *MCT1* ^+/+^ mice when fed HFD. Food intake was examined in *MCT1* ^+/**−**^ and *MCT1* ^+/+^ mice but under NC, no significant difference was observed (data not shown). Under HFD however, distinct patterns emerged as *MCT1* ^+/+^ mice started to gain weight at a higher rate than *MCT1* ^+/**−**^ mice from the third week under HFD (Fig. 6A). Reported as cumulated body weight gain, it can be seen that the difference becomes highly significant from the third week under HFD (Fig. 6B). When food intake is plotted, 2 phases can be distinguished (Fig. 6C). A rapid decrease in food intake occurs in the first 2 weeks, followed by stabilization in the amount of food ingested. *MCT1* ^+/**−**^ mice maintained on HFD exhibited from the third week onward a lower food intake than *MCT1* ^+/+^ mice. The difference can be even better appreciated when data are reported as cumulated food intake, clearly showing that *MCT1* ^+/**−**^ mice had a lower quantity of ingested food compared to *MCT1* ^+/+^ mice (Fig. 6D). Interestingly, the appearance of food intake difference correlates with the divergence of body weight between *MCT1* ^+/**−**^ and *MCT1* ^+/+^ mice (compare Fig. 6A with 6C). Intestinal absorption also determines the amount of energy intake by the organism. To evaluate intestinal absorption, stool energy content for both *MCT1* ^+/**−**^ and *MCT1* ^+/+^ mice under HFD was determined. Indeed, *MCT1* ^+/**−**^ mice exhibited a 9.6% higher stool energy content than *MCT1* ^+/+^ mice (17596±323 vs. 16055±264 J/g of stool, *P*<0.01). Thus, both hypophagia and reduced energy assimilation are key contributors to the resistance to diet-induced obesity in HFD-fed *MCT1* ^+/**−**^ mice.

**Figure 7:**
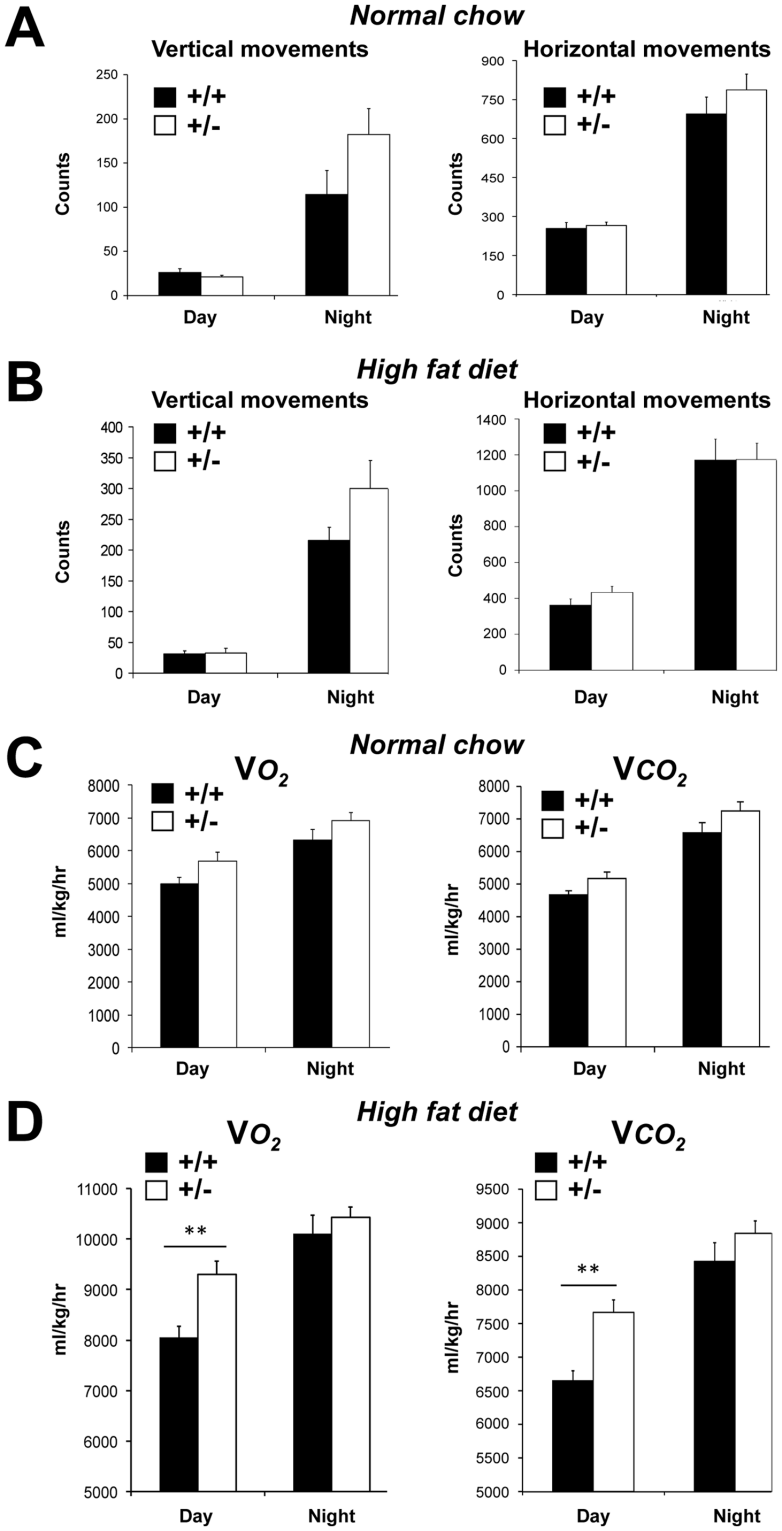
Locomotor activity and indirect calorimetry for *MCT1* ^+/+^ and *MCT1* ^+/−^ mice fed a normal chow or high fat diet. (**A**) Vertical and horizontal movements of *MCT1* ^+/+^ and *MCT1* ^+/−^ mice under normal chow. (**B**) Vertical and horizontal movements of *MCT1* ^+/+^ and *MCT1* ^+/−^ mice under high fat diet. Filled bars, *MCT1* ^+/+^ mice; open bars, *MCT1* ^+/−^ mice. (**C**) O_2_ consumption and CO_2_ production of *MCT1* ^+/+^ and *MCT1* ^+/−^ mice fed a normal chow. (**D**) O_2_ consumption and CO_2_ production of *MCT1* ^+/+^ and *MCT1* ^+/−^ mice fed a high fat diet. V*O_2_*, oxygen consumption ; V*CO_2_*, carbon dioxide production. Filled bars, *MCT1* ^+/+^ mice; open bars, *MCT1* ^+/−^ mice. Results represent mean ± SEM, n = 5-8. Asterisks represent a statistically significant difference between *MCT1* ^+/+^ and *MCT1* ^+/−^ mice evaluated using a Student’s *t*-test. **P<0.01.

Energy expenditure components include the metabolic rate and physical activity. Energy homeostasis was assessed using the Comprehensive Laboratory Animal Monitoring System (CLAMS). Locomotor activity was evaluated by recording both horizontal and vertical movements over a period of 3 days and by reporting the cumulated data for the diurnal and nocturnal periods. No significant difference was observed between *MCT1* ^+/**−**^ and *MCT1* ^+/+^ mice when fed either NC or HFD (Fig. 7A, B). Indirect calorimetry was used to assess the metabolic rate by measuring both oxygen consumption (VO_2_) and carbon dioxide production (VCO_2_) over a period of 3 days. No significant difference in either oxygen consumption or carbon dioxide production was observed between *MCT1* ^+/**−**^ and *MCT1* ^+/+^ mice fed NC (Fig. 7C). When fed HFD for 9 weeks however, *MCT1* ^+/**−**^ mice consumed 15% more oxygen (VO_2_  =  9300±264 vs. 8052±221 ml.kg^–1^.h^–1^ respectively; *P*<0.01) and consequently produced 15% more carbon dioxide VCO_2_ = 7662±192 vs. 6646±150 ml.kg^–1^.h^–1^ respectively; *P*<0.01) than *MCT1* ^+/+^ mice during daytime, but not during nighttime (Fig. 7D). In addition, respiratory quotients were calculated in all cases. Under normal chow, no significant difference was observed between *MCT1* ^+/**−**^ and *MCT1* ^+/+^ mice during daytime (0.92±0.01 vs. 0.94±0.01 respectively; *P* = 0.32) or nighttime (1.05±0.01 vs. 1.04±x10**^−^** ^5^ respectively; *P* = 0.376). When animals were fed HFD, respiratory quotient values decreased for all conditions, but no significant difference was observed between *MCT1* ^+/**−**^ and *MCT1* ^+/+^ mice during daytime (0.83±0.01 vs. 0. 83±0.01 respectively; *P* = 0.91) while a very small but significant difference was detected during nighttime (0.85±1×10**^−^** ^5^ vs. 0.83±1×10**^−^** ^5^ respectively; *P*<0.05, corresponding to 49% vs. 42% of carbohydrate consumption).

### Attenuated alterations in some circulating hormone levels and in hepatic lipid metabolism are distinctive features of HFD-fed *MCT1^+/^* ^−^ mice

In order to gain further insight about the putative molecular mechanisms that could explain the observed phenotype in *MCT1^+/^* ^**−**^ mice, the plasma content of several metabolites and hormones was analyzed in both *MCT1^+/^* ^**−**^ and *MCT1^+/+^* mice under either normal chow or HFD for four months (Table 3). Under normal chow, no significant differences were observed for most parameters tested, except for lactate that was higher in *MCT1^+/^* ^−^ mice and triglycerides that were lower in *MCT1^+/^* ^−^ mice. Under HFD however, several parameters were differentially affected. Triglycerides were still lower in *MCT1^+/^* ^−^ mice although the difference did not reach significance. The plasma level of the enzyme alanine aminotransferase (ALAT; a marker of hepatocellular injury) was lower in *MCT1^+/^* ^−^ mice. Interestingly, the levels of insulin and leptin, which were increased in *MCT1^+/+^* mice under HFD compared to normal chow, were significantly lower in *MCT1^+/^* ^−^ mice under HFD. When the HOMA (homeostasis model assessment) index was calculated (an indication of insulin resistance), a similar observation was made with a significantly lower value in *MCT1^+/^* ^−^ mice under HFD compared to *MCT1^+/+^* mice.

**Table 3:** Plasma biochemical profile of MCT1^+/+^ and MCT1^+/−^ mice fed a normal chow or a high fat diet.

	Normal chow		High Fat Diet	
Plasma parameter	MCT1^+/+^ (5)	MCT1^+/−^ (7)	MCT1^+/+^ (7)	MCT1^+/−^ (12)
Glycemia (mg/dL)	161.8±6.3	140.0±5.7	177.5±14.8	162.3±4.5
Ketone bodies (mM)	0.22±0.02	0.21±0.03	0.31±0.05	0.31±0.03
Lactate (mM)	5.1±0.5	7.0±0.7*	4.5±0.5	3.7±0.3
Cholesterol (mM)	2.7±0.2	2.5±0.1	4.1±0.6	3.6±0.3
HDL (mM)	1.5±0.1	1.6±0.1	2.6±0.4	2.4±0.2
LDL (mM)	0.25±0.06	0.23±0.02	0.34±0.06	0.33±0.03
Triglycerides (mM)	1.8±0.4	1.1±0.1*	1.4±0.1	0.92±0.07
FFA (mM)	0.41±0.05	0.39±0.05	0.72±0.08	0.58±0.06
Lipase (mM)	53.7±3.5	52.4±4.3	53.1±2.1	71.4±9.3
LDH (U/L)	1381.6±267.1	1128.3±228.8	1940.3±225.5	1631.8±121.2
ALAT (U/L)	36.6±2.7	50.7±6.9	150.3±39.2	83.7±12.7*
ASAT (U/L)	213.8±46.7	210.5±51.7	534.9±135.1	469.2±46.5
Ghrelin (ng/mL)	2.71±0.66	1.39±0.42	1.57±0.25	2.20±0.40
Leptin (ng/mL)	0.70±0.08	0.74±0.19	15.58±1.99	4.72±0.72***
Insulin (ng/mL)	1.469±0.390	1.986±0.482	6.400±1.848	2.167±0.496*
HOMA index	0.744±0.207	1.046±0.263	3.097±0.888	0.895±0.233**

Results are expressed as mean ± SEM; values within parentheses indicate number of mice. *P<0.05, **P<0.01, ***P<0.001 vs. *MCT1^+/+^* on the same diet with two-way ANOVA followed by Bonferroni post hoc test. HDL, High density lipoprotein; LDL, Low density lipoprotein; FFA, Free fatty acids; LDH, Lactate dehydrogenase; ALAT, Alanine aminotransferase; ASAT, Aspartate aminotransferase; HOMA, Homeostasis model assessment.

**Figure 8:**
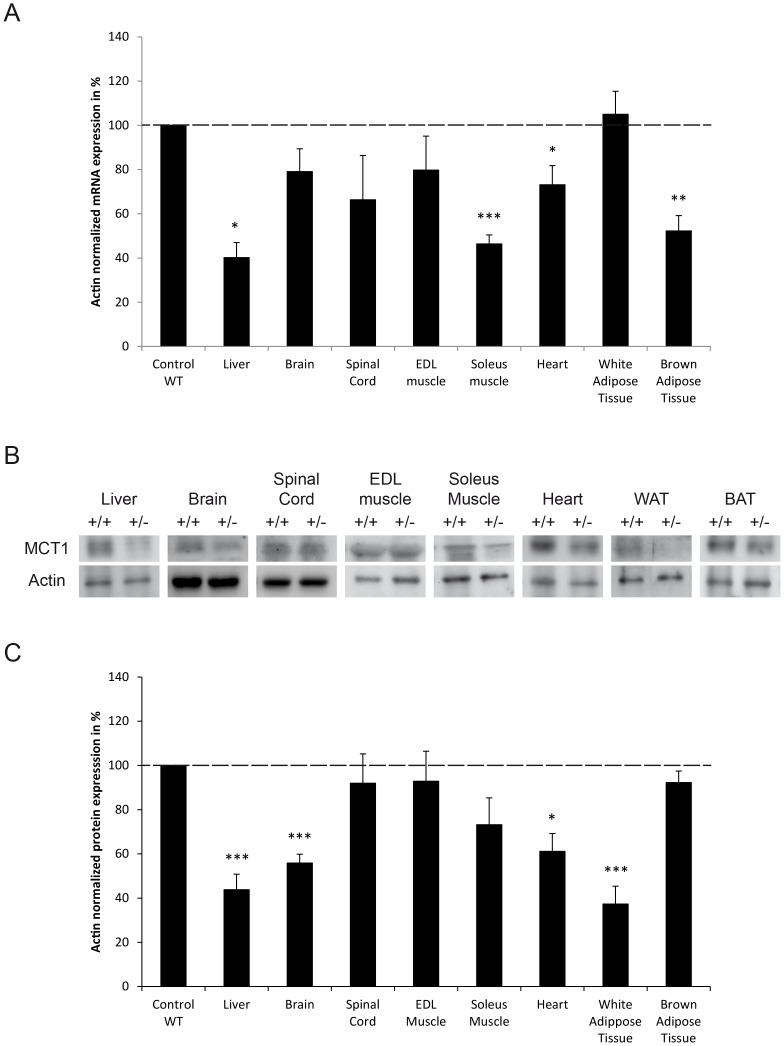
MCT1 mRNA and protein expression levels in tissues from *MCT1* ^+/+^ vs. *MCT1* ^+/−^ mice following three months of high fat diet (A) Quantitative RT-PCR analysis of relative MCT1 mRNA levels in tissues collected from *MCT1^+/−^* and *MCT1^+/+^* mice. Values are expressed as percentage and represent the mean normalized MCT1 mRNA level in *MCT1^+/^* ^−^ mouse tissue compared to the mean normalized MCT1 mRNA level in *MCT1^+/+^* mouse tissue ± SEM, n = 4 (*MCT1^+/+^*) and 7 (*MCT1^+/^* ^−^). ß-actin mRNA was used as reference. Statistical analysis was performed using the Student’s t-test. Asterisks indicate a significant difference between heterozygote and wildtype tissues. *P<0.05, **P<0.01, ***P<0.001. (**B**) Representative western blot performed on protein extracts from selected tissues of *MCT1^+/+^* and *MCT1^+/^* ^−^ mice. ß-actin was used as reference for quantification. (**C**) Quantitative analysis of MCT1 protein expression. Values are expressed as percentage, and represent the mean normalized MCT1 protein level of *MCT1^+/^* ^−^ mice compared to the mean normalized MCT1 protein level for *MCT1^+/+^* mice ± SEM, n = 4 (*MCT1^+/+^*) and 7 (*MCT1^+/^* ^−^). Statistical analysis was performed using the Student’s *t*-test. Asterisks indicate a significant difference between heterozygote and wildtype tissues. *P<0.05, **P<0.01, ***P<0.001.

**Figure 9:**
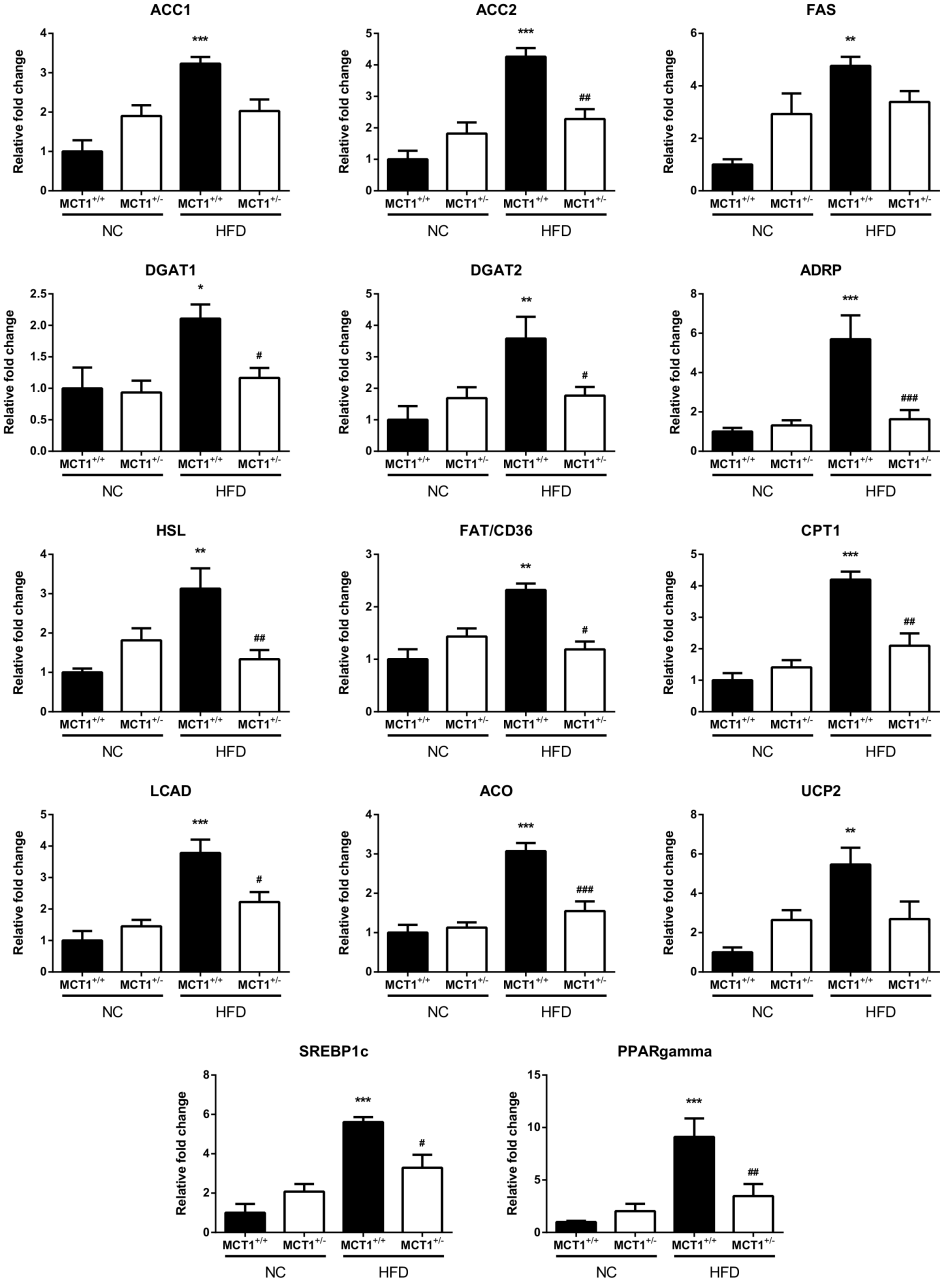
Relative liver mRNA expression levels for various genes involved in lipid metabolism from *MCT1^+/−^* and *MCT1^+/+^* mice fed either normal chow or high fat diet. Quantitative RT-PCR analysis of relative mRNA levels for several genes related to lipid metabolism in liver collected from *MCT1^+/^* ^−^ and *MCT1^+/+^* mice either fed normal chow or high fat diet for three months. Values represent the mean normalized mRNA level relative to the level found in *MCT1^+/^* ^−^ mouse liver fed normal chow (the former given a value of one) ± SEM, n = 5 (normal chow, *MCT1^+/+^*), 7 (normal chow, *MCT1^+/^* ^−^) 4 (HFD, *MCT1^+/+^*), 8 (HFD, *MCT1^+/^* ^−^). Statistical analysis was performed using a two-way ANOVA followed by a Tukey HSD *post hoc* test. Asterisks indicate a significant difference between liver samples from wildtype animals under HFD vs. normal chow. *P<0.05, **P<0.01, ***P<0.001. Sharp signs indicate a significant difference between liver samples from heterozygote animals under HFD vs. wildtype animals under HFD. ^#^ *P*<0.05, ^##^ *P*<0.01, ^###^ *P*<0.001. ACC, Acetyl-CoA carboxylase; ACO, Acyl-CoA-oxidase; ADRP, Adipose differentiation-related protein; CPT, Carnitine palmitoyltransferase; DGAT, diacylglycerol acyltransferase; FAS, Fatty acid synthase; FAT/CD36, Fatty acid translocase; HSL, Hormone-sensitive lipase; LCAD, Long chain acyl-CoA dehydrogenase; PPAR, Peroxisome proliferator-activated receptor; SREBP, Sterol regulatory element-binding protein; UCP, Uncoupling protein.

Although differences of MCT1 expression were observed in several organs and tissues between *MCT1^+/^* ^−^ and *MCT1^+/+^* mice fed a normal chow (Fig. 2A-C), this pattern might not be the same when animals are exposed to HFD for a prolonged period. For this reason, both MCT1 mRNA and protein expression was re-examined in the same organs and tissues but after three months under HFD (Fig. 8). Indeed, although some features remained (e.g. lower expression in *MCT1^+/^* ^−^ brain and heart), new ones emerged. This is the case for the liver that did not exhibited any difference under normal chow but in which MCT1 expression became lower in *MCT1^+/^* ^−^ mice compared to *MCT1^+/+^* mice under HFD, both at the mRNA (Fig. 8A) and at the protein level (Fig. 8B, C). These observations prompted us to examine in the liver the expression of various genes involved in lipid metabolism between *MCT1^+/^* ^−^ and *MCT1^+/+^* mice under either normal chow or HFD for three months (Fig. 9). Genes could be grouped into different categories, being involved either in lipid synthesis (i.e. ACC1, ACC2, FAS, DGAT1, DGAT2), storage (i.e. ADRP), release (i.e. HSL), transport (i.e. FAT/CD36, CPT1), oxidation (i.e. LCAD, ACO, UCP2) or in regulation of lipid metabolism (i.e. SREBP1c, PPARgamma). Quite remarkably, three months under high fat diet led to an enhancement of mRNA expression for all genes involved in lipid metabolism investigated in the liver of *MCT1^+/+^* mice. In contrast, in the liver of *MCT1^+/^* ^−^ mice, either no enhancement or a much reduced level of mRNA expression compared to *MCT1^+/+^* mice was observed for the same genes following HFD.

## Discussion

The *MCT1* null mouse exhibits an embryonic lethal phenotype, restricting possibilities to study MCT1 functions in this species. Interestingly, an MCT1 ortholog called *Silnoon* has been identified in *Drosophila melanogaster* and various loss-of-function mutants have been generated [24]. Similarly to our *MCT1* null mouse, the homozygous fly mutant for *Silnoon* exhibited early larval lethality. This observation suggests that the use of monocarboxylates is crucial for early growth and development in different species. It is noteworthy that abundant placental MCT1 expression was reported, as early as gestational day 11.5 in mice [25]. In this context, placental MCT1 expression might play a key role in the transfer of monocarboxylates from the maternal circulation and ensure proper substrate supply for embryonic development. It is purported that absence of placental MCT1 expression might be a cause of early lethality.

In the absence of a viable null mutant, the *MCT1^+/^* ^−^ mouse was further investigated. The phenotype observed was mild with no marked morphological or behavioural alteration. Similarly, no obvious changes were observed in any tissue that was examined histologically. The explanation may lie in the extent of the reduction of MCT1 expression found in the different tissues analyzed that never exceeded 50% at the protein level. These levels of expression might be sufficient to maintain proper function in most tissues and organs under normal diet. For example, MCT1 knockdown in cultured astrocytes reduced lactate transport but not stimulated lactate release [26]. However, we can not exclude that other monocarboxylate transporters might have compensated for decreased MCT1 expression.

The C57BL/6J mouse strain is sensitive to diet-induced obesity and represents a good model to study the underlying mechanisms leading to this condition [27]. Such a vulnerability to the development of obesity could also be exploited to identify specific genes that give rise to this susceptibility. Indeed, invalidation of a subset of genes has been shown to confer at least partial resistance to diet-induced obesity, e.g. the µ opioid receptor [28], the retinoblastoma protein [29], the peroxisome proliferating-activated receptor gamma [30], the very-long-chain fatty acid elongase [31] or the CB1 cannabinoid receptor [32]. These genes have been qualified of “thrifty genes” as they promote the storage of energy reserves in time of food abundance, an important function from an evolutionary point of view. The *mct1* gene appears to belong to this group but represents a new protein class, i.e. nutrient transporters. In addition, the haploinsufficiency of the *mct1* gene in regard to body weight regulation is noteworthy. Indeed, it is useful to point out that body weight regulation is also influenced in the case of haploinsufficiency of genes involved in the development of monogenetic obesity such as the leptin gene or the POMC gene [33], [34]. Accordingly, such a characteristic seems to further emphasize the critical role played by these genes in energy homeostasis.

Our investigation to determine which parameter(s) of the energy balance influenced by MCT1 expression affect(s) body weight gain under HFD has revealed alterations in energy intake. Although no difference in food intake was observed between *MCT1^+/^* ^−^ and *MCT1^+/+^* mice when fed NC, a more important reduction in food intake occurred in *MCT1^+/^* ^−^ mice when switched to HFD. Control of food intake takes place in the lateral hypothalamus where the presence of fuel-sensing neurons capable to modify their firing patterns in response not only to glucose but also to monocarboxylates has been reported [5]. Moreover, it was demonstrated that such neurons express the monocarboxylate transporter MCT1 [5]. One possible explanation for the observed food intake adaptations is that changes in MCT1 expression levels on some hypothalamic neurons alters the sensitivity of the circuit controlling food intake to specific peripheral signals (e.g. insulin, leptin or ghrelin), whose levels vary in response to HFD. Indeed, plasma insulin and leptin levels were reduced in *MCT1^+/^* ^−^ mice compared to *MCT1^+/+^* mice, suggesting that sensitivity to these hormones was likely preserved in *MCT1^+/^* ^−^ mice, in contrast to *MCT1^+/+^* mice. Interestingly, *Drosophila* mutants for *Silnoon* (the MCT1 ortholog) displayed halted food intake [24], which indeed suggests that MCT1 expression is critical for this function. In parallel, the higher caloric content of feces observed in *MCT1^+/^* ^−^ mice indicates reduced intestinal absorption of some nutrients. MCT1 is differentially expressed in various parts of the gastrointestinal tract [19] and it was observed that MCT1 expression on the luminal membrane of intestinal epithelial cells is critical for the transport of short-chain fatty acids such as butyrate [35]. Short-chain fatty acids were shown not only to serve as precursors for lipogenesis [36], but in intestinal cells they also downregulate expression of genes involved in lipid metabolism, e.g. cholesterol synthesis [37]. Thus, a reduction in intestinal MCT1 expression might impact on the capacity of the intestine to properly extract short-chain fatty acids and might as well modify intestinal lipid metabolism.

Diet-induced obesity in mice is often characterized by glucose intolerance, insulin resistance, elevated insulinemia as well as leptinemia associated with increased fat mass [27], [38]. Many treatments aiming at reducing or preventing the development of obesity tend to normalize these different parameters [39], [40]. Similarly, transgenic mice identified to resist to the development of diet-induced obesity usually exhibit attenuation or even absence of these metabolic dysregulations [28], [29], [31], [32]. It seems also to be the case of HFD fed *MCT1^+/^* ^−^ mice. Thus, a greater glucose tolerance as well as higher insulin sensitivity could be evidenced in HFD fed *MCT1^+/^* ^−^ mice compared to HFD fed *MCT1^+/+^* mice. Along with the improvement of insulinemia, the insulin resistance present in HFD fed *MCT1^+/+^* mice was not detected in HFD fed *MCT1^+/^* ^−^ mice. In parallel, fat mass was significantly reduced while leptinemia returned to almost normal values. Thus, it appears that haploinsufficiency of the *mct1* gene, in parallel of reducing fat mass accumulation, prevents the emergence of the main characteristic metabolic dysregulations associated with obesity. Whether the reduction in fat mass is the sole factor explaining the normal glucose tolerance, the absence of insulin resistance as well as almost normal insulinemia and leptinemia remains to be demonstrated. However, preliminary results about the relationship between fat mass and insulin resistance (Fig. S3) suggest that under high fat diet, others factors might also contribute to the metabolic dysregulations observed. Indeed, it was previously shown that factors such as inflammation, lipotoxicity or gut microbial dysbiosis can also participate to the development of insulin resistance, in association or not with increased fat mass [41].

Prevention of hepatic steatosis and reduction in adiposity are important features of *MCT1^+/^* ^−^ mice under HFD, and these are common traits among animal models exhibiting resistance to diet-induced obesity. Modifications in fat and/or glucose metabolism, as documented by changes in expression of genes related to their metabolism, often provide an explanation for the prevention of the energy imbalance that leads to the observed phenotypic characteristics in diet-induced obese animals. Thus, an enhancement of fatty acid oxidation occurring in the liver [29] and/or in muscle [28], [29], a decrease of lipogenesis in the liver [31], or alterations of lipogenesis/lipolysis in the adipose tissue [42], [43] are common findings in these models. In contrast to the upregulation of genes involved in lipid metabolism in the liver of HFD fed *MCT1^+/+^* mice, an observation in agreement with published observations [44], the expression of the same genes was relatively unaffected in the liver of HFD fed *MCT1^+/^* ^−^ mice. Thus, no apparent enhancement in hepatic lipid synthesis and storage capacity is taking place in *MCT1^+/^* ^−^ mice exposed to HFD. These findings seem corroborated by the tendency toward a reduction in plasma triglycerides that was detected in HFD fed *MCT1^+/^* ^−^ mice compared to *MCT1^+/+^* mice. Thus, a lack of enhanced lipid synthesis and storage capacity in the liver could be a plausible explanation for the prevention of hepatic steatosis and obesity, as observed in several transgenic mouse models [45]. Indeed, it was previously shown that inhibition or altered expression of some key proteins involved in lipogenesis can prevent hepatic steatosis and dyslipidemia [31], [46]-[48]. In parallel, no enhancement in the expression of genes involved in fatty acid oxidation could be evidenced in the liver of HFD fed *MCT1^+/^* ^−^ mice, arguing against an increase in hepatic ß-oxidation. Hence, hepatic fatty acid oxidation does not appear to contribute to the prevention of hepatic steatosis, and *a fortiori* to overall reduced fat mass in HFD fed *MCT1^+/^* ^−^ mice. The question arises about the role of MCT1 *per se* in the liver in relation with adaptation of lipid metabolism. Although no difference in MCT1 expression at the mRNA and protein levels could be detected in the liver between *MCT1^+/^* ^−^ and *MCT1^+/+^* mice under normal chow, both MCT1 mRNA and protein levels were significantly lower in the liver of *MCT1^+/^* ^−^ mice fed HFD vs. *MCT1^+/+^* mice fed HFD. It is conceivable then that MCT1 expression in the liver might have influenced hepatic lipid metabolism. However, no significant difference in the plasma levels of MCT1 substrates such as lactate and ketone bodies between the two genotypes could be observed, which seems to rule out a reduced capacity to use or produce these substrates, although we can not exclude a reallocation of these substrates to tissues with a different MCT subtype expression profile. Nevertheless, our results revealed that reduced hepatic MCT1 expression under HFD interfered with the adaptive metabolic response of the liver to HFD towards lipids, although the mechanism remains for the moment unclear.

Putative alterations in hepatic liver metabolism suggested by the uncovered gene expression pattern in animals fed HFD may not entirely explain the overall reduction in fat mass. Other mechanisms must be postulated to fully account for this specific feature. Possible alterations in lipid/glucose metabolism could be present as well in other tissues including muscle and adipose tissue. For example, an increase in muscle energy substrate oxidation could account for the observed enhancement in daytime metabolic rate. If it is the case however, it does not appear to be due to a shift in the type of substrates oxidized (i.e. lipids at the expense of carbohydrates) based on the calculated respiratory quotients, but it should rather represents simply an overall enhanced oxidation rate. Increased muscle fatty acid oxidation was previously implicated in the prevention of diet-induced fat mass accumulation [28], [29], [49]. Similarly, we can not exclude that some aspects of adipose tissue metabolism are affected, including lipid storage and lipolysis. Indeed, although no difference in MCT1 expression could be detected between *MCT1^+/^* ^−^ and *MCT1^+/+^* mice under normal chow in white adipose tissue, MCT1 protein levels were significantly lower in HFD fed *MCT1^+/^* ^−^ mice vs. HFD fed *MCT1^+/+^* mice. Further investigations in both muscle and adipose tissue will be necessary to assess such possible metabolic alterations.

In summary, the present work demonstrates that the partial invalidation of the *mct1* gene leads to specific metabolic and behavioral adaptations preventing the development of diet-induced obesity, hepatic steatosis and insulin resistance in mice. Further studies will be necessary to shed light on the precise mechanisms by which a reduction in MCT1 expression in specific tissues give rise to the described phenotypical characteristics. However, our study already provides a proof-of-concept that the MCT1 transporter represents a new therapeutic target to prevent and/or treat obesity.

## Supporting Information

Figure S1 **Comparative histological analysis of various tissues from** ***MCT1*** **^+/+^ and** ***MCT1*** **^+/^** ^−^ **mice fed a normal chow.** BAT, brown adipose tissue. Calibration bar, 100 µm.
(TIF)

Figure S2 **Comparative histological analysis of various tissues from** ***MCT1*** **^+/+^ and** ***MCT1*** **^+/^** ^−^ **mice fed a high fat diet.** BAT, brown adipose tissue. Calibration bars, 20 or 100 µm.
(TIF)

Figure S3 **Relationship between the percentage of fat mass and the degree of insulin resistance (as evaluated by the HOMA index) in both** ***MCT1^+/+^*** **and** ***MCT1^+/^*** ^−^ **mice fed either a normal chow or a high fat diet.** Filled circle, *MCT1^+/+^* mouse fed normal chow ; filled triangle, *MCT1^+/^* ^−^ mouse fed normal chow ; Open circle, *MCT1^+/+^* mouse fed high fat diet; Open triangle, *MCT1^+/^* ^−^ mouse fed high fat diet. NC, normal chow diet ; HFD, high fat diet.
(TIF)

Table S1 **SHIRPA screening test of MCT1^+/+^ and MCT^+/^** ^−^ **.**
(TIF)
