# Supplementary figures and images for: Resistance to Diet-Induced Obesity and Associated Metabolic Perturbations in Haploinsufficient Monocarboxylate Transporter 1 Mice
Source: PLoS One. 2013 Dec 18;8(12):e82505. doi: 10.1371/journal.pone.0082505 (PMC3867350; doi:10.1371/journal.pone.0082505)

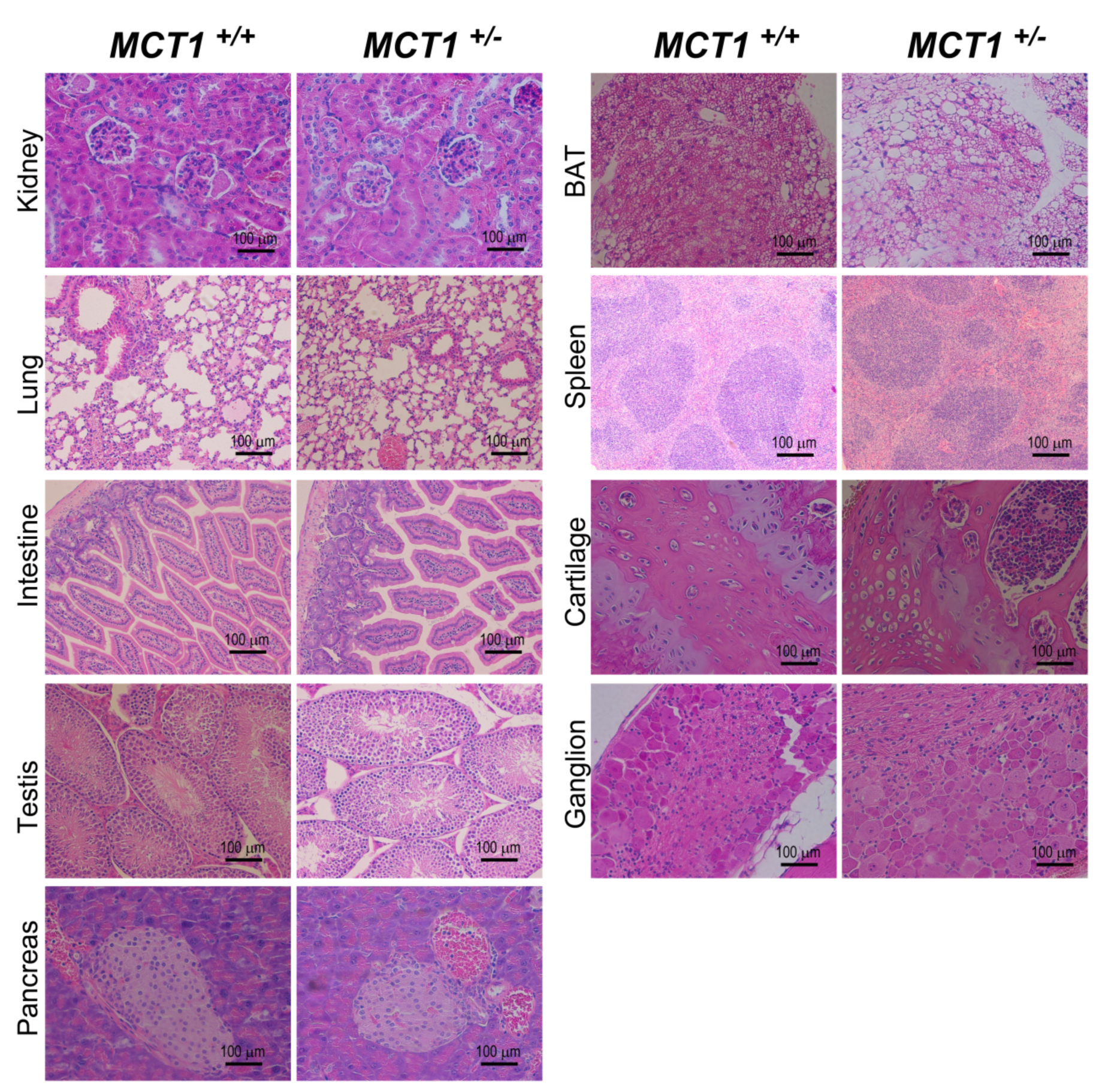

Supplement: Figure S1 — Comparative histological analysis of various tissues from MCT1 +/+ and MCT1 +/ − mice fed a normal chow. BAT, brown adipose tissue. Calibration bar, 100 µm. (TIF) [file pone.0082505.s001.tif]

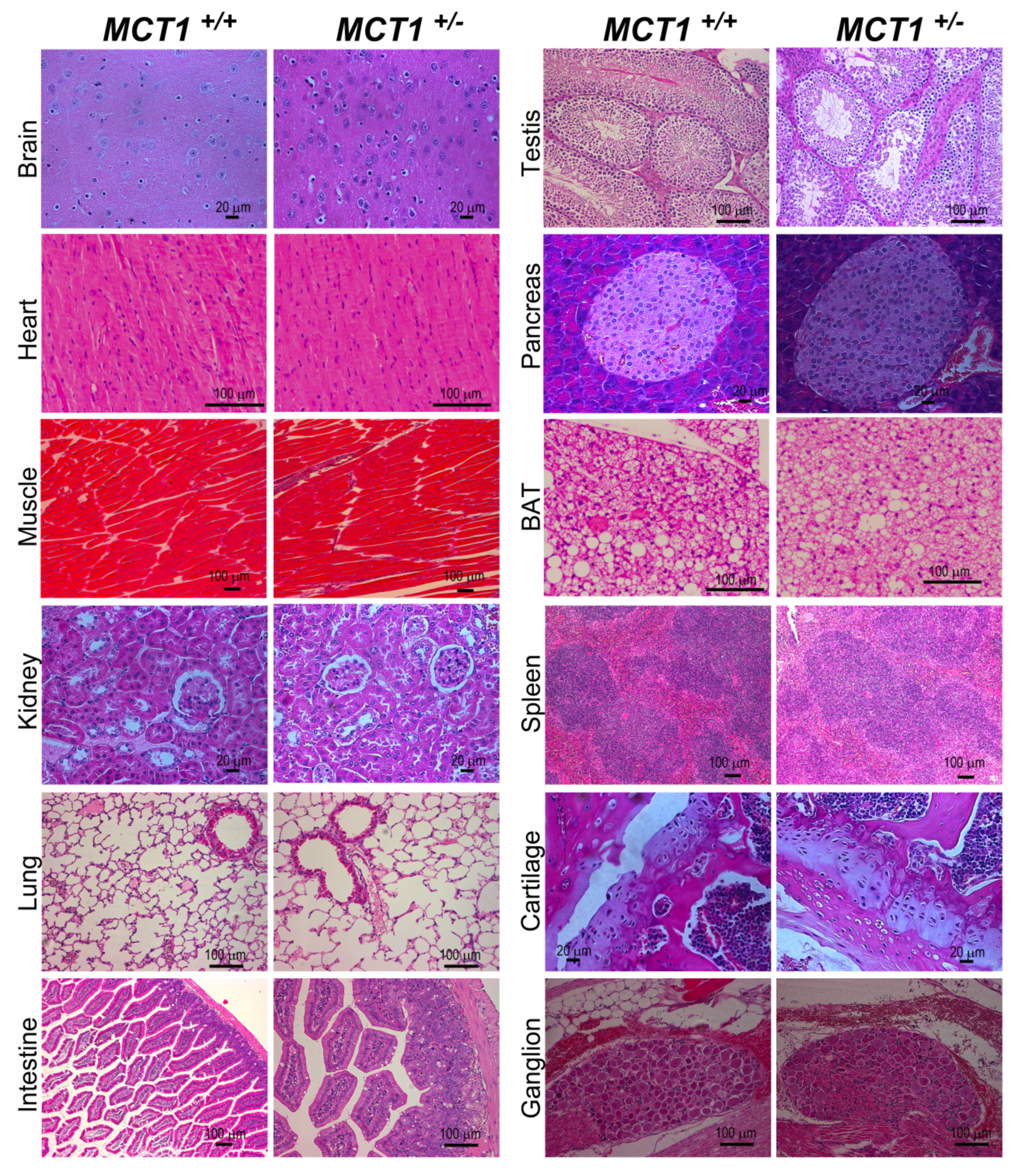

Supplement: Figure S2 — Comparative histological analysis of various tissues from MCT1 +/+ and MCT1 +/ − mice fed a high fat diet. BAT, brown adipose tissue. Calibration bars, 20 or 100 µm. (TIF) [file pone.0082505.s002.tif]

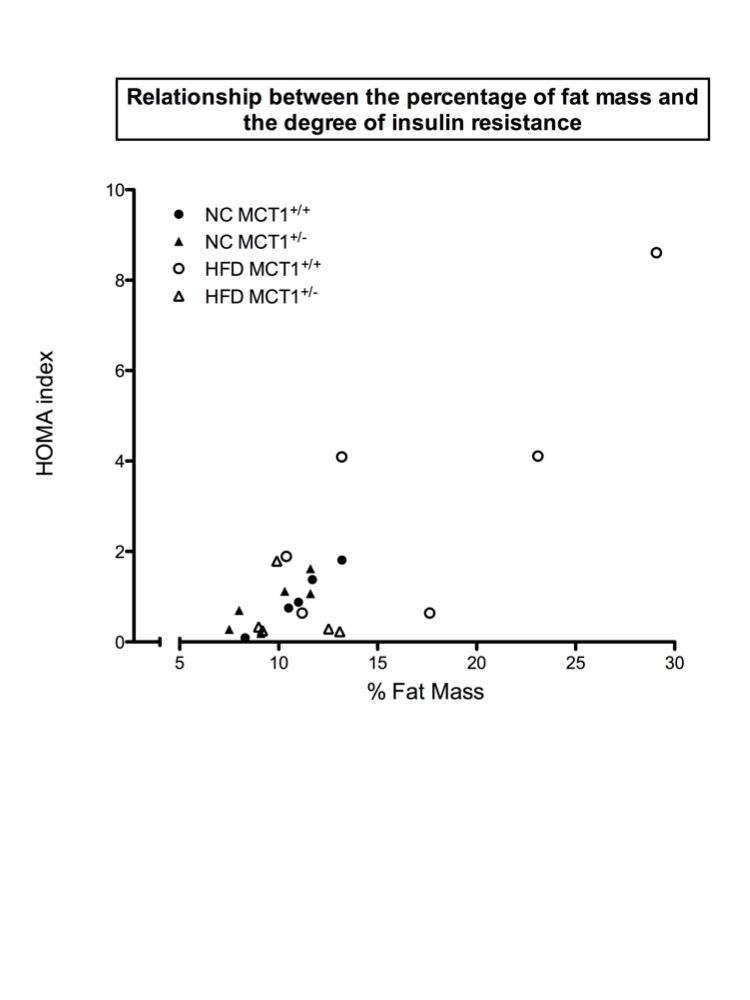

Supplement: Figure S3 — Relationship between the percentage of fat mass and the degree of insulin resistance (as evaluated by the HOMA index) in both MCT1+/+ and MCT1+/ − mice fed either a normal chow or a high fat diet. Filled circle, MCT1+/+ mouse fed normal chow ; filled triangle, MCT1+/ − mouse fed normal chow ; Open circle, MCT1+/+ mouse fed high fat diet; Open triangle, MCT1+/ − mouse fed high fat diet. NC, normal chow diet ; HFD, high fat diet. (TIF) [file pone.0082505.s003.tif]

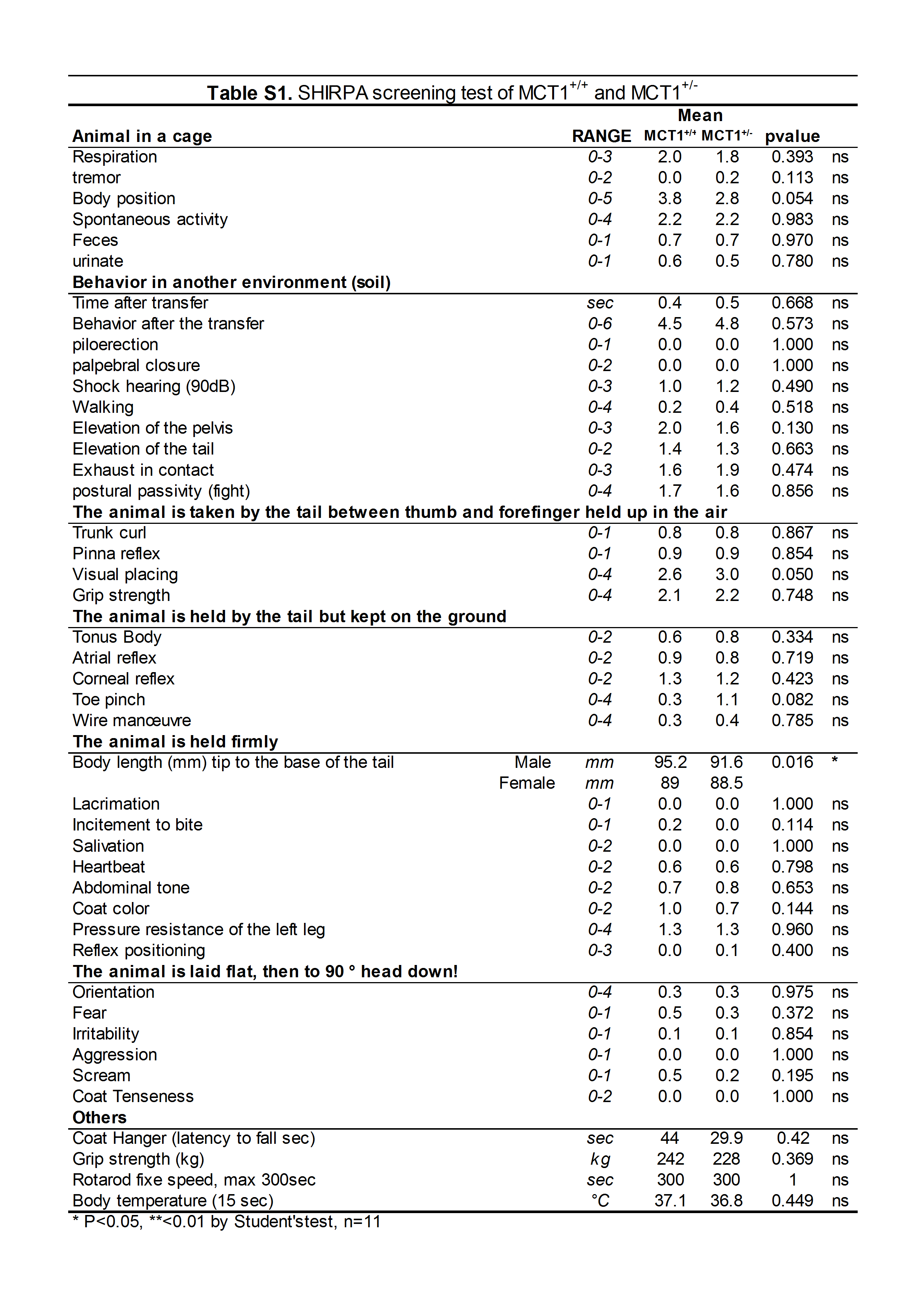

Supplement: Table S1 — SHIRPA screening test of MCT1+/+ and MCT+/ − . (TIF) [file pone.0082505.s004.tif]
